# Proximity labeling identifies a repertoire of site-specific R-loop modulators

**DOI:** 10.1038/s41467-021-27722-6

**Published:** 2022-01-10

**Authors:** Qingqing Yan, Phillip Wulfridge, John Doherty, Jose L. Fernandez-Luna, Pedro J. Real, Hsin-Yao Tang, Kavitha Sarma

**Affiliations:** 1 Gene Expression and Regulation Program, The Wistar Institute, Philadelphia, PA 19104 USA; 2 Epigenetics Institute, University of Pennsylvania, Philadelphia, PA 19104 USA; 3 Genetics Unit, Hospital Valdecilla, 39008 Santander, Spain; 4 Instituto de Investigación Valdecilla (IDIVAL), 39012 Santander, Spain; 5 Gene Regulation, Stem Cells and Development Group, Department of Genomic Oncology, GENYO: Centre for Genomics and Oncological Research-Pfizer, University of Granada, Junta de Andalucía, PTS, 18016 Granada, Spain; 6 Department of Biochemistry and Molecular Biology I, Faculty of Science, University of Granada, 18016 Granada, Spain; 7 The Wistar Institute, Philadelphia, PA 19104 USA

**Keywords:** Proteomics, RNA metabolism

## Abstract

R-loops are three-stranded nucleic acid structures that accumulate on chromatin in neurological diseases and cancers and contribute to genome instability. Using a proximity-dependent labeling system, we identified distinct classes of proteins that regulate R-loops in vivo through different mechanisms. We show that ATRX suppresses R-loops by interacting with RNAs and preventing R-loop formation. Our proteomics screen also discovered an unexpected enrichment for proteins containing zinc fingers and homeodomains. One of the most consistently enriched proteins was activity-dependent neuroprotective protein (ADNP), which is frequently mutated in ASD and causal in ADNP syndrome. We find that ADNP resolves R-loops in vitro and that it is necessary to suppress R-loops in vivo at its genomic targets. Furthermore, deletion of the ADNP homeodomain severely diminishes R-loop resolution activity in vitro, results in R-loop accumulation at ADNP targets, and compromises neuronal differentiation. Notably, patient-derived human induced pluripotent stem cells that contain an ADNP syndrome-causing mutation exhibit R-loop and CTCF accumulation at ADNP targets. Our findings point to a specific role for ADNP-mediated R-loop resolution in physiological and pathological neuronal function and, more broadly, to a role for zinc finger and homeodomain proteins in R-loop regulation, with important implications for developmental disorders and cancers.

## Abstract (web summary)

R-loops are three-stranded nucleic acid structures that contribute to genome instability and accumulate in neurological diseases. Here the authors identify R-loop proximal factors, which are enriched for zinc finger and homeodomain proteins, including activity-dependent neuroprotective protein (ADNP). ADNP plays a role in R-loop resolution and loss-of-function leads to R-loop accumulation.

## Introduction

Chemical and structural deregulation of chromatin is implicated in neurodevelopmental and neurodegenerative disorders, cancers, and other diseases. A poorly understood chromatin structure that is associated with several neurodevelopmental disorders is the R-loop^1–3^. R-loops are three-stranded nucleic acid structures comprising a DNA:RNA hybrid and a displaced single-stranded DNA (ssDNA)^4^ that primarily occur as a consequence of transcription^5,6^. R-loops can be stabilized when the displaced ssDNA folds into a G quadruplex (G4) structure. R-loops have important regulatory roles in the nucleus^7^ and alterations in R-loop levels have effects on transcription and DNA repair^1,2^. While temporary R-loop formation is essential to important physiological processes, such as immunoglobulin class switch recombination^8^, their persistence is often associated with adverse outcomes. For example, persistent at telomeres are associated with compromised genome integrity^7,9^. Aberrant R-loops also form at genomic regions associated with nucleotide repeat expansion disorders such as Fragile X syndrome (FXS)^10^ and Friedreich ataxia (FRDA)^11^. The formation of R-loops at expanded repeats in FXS and FRDA is proposed to alter chromatin modifications and inhibit transcription of the *FMR1* and *FXN* genes, respectively^11^, pointing to a pathogenic role for these chromatin structures.

R-loops are thought to be resolved mainly by helicases that unwind the DNA:RNA hybrid or the G4 structures in ssDNA. Helicases implicated in R-loop regulation include SETX, DDX5, DDX39B, and ATRX^12–15^. In addition, ribonuclease H (RNase H) enzymes specifically degrade the RNA within DNA:RNA hybrids to restore dsDNA. Eukaryotes contain two RNase H enzymes, RNase H1 and RNase H2, with distinct substrate preferences and cell cycle-specific roles^16^. Other proteins that can influence R-loop levels include topoisomerases that relieve topological stress during transcription and replication^17,18^; proteins that regulate helicase localization^19^ or stimulate RNase H activity^20^; and ssDNA binding proteins that can stabilize R-loops by preventing the reannealing of DNA strands^21^. These factors work in concert to preserve biologically important R-loops while ensuring that harmful R-loops are quickly resolved.

The dynamic nature of R-loops makes the identification of transient interactors challenging. Two recent studies have used unbiased proteomics to screen for R-loop regulators. In one approach, the S9.6 monoclonal antibody that recognizes DNA:RNA hybrids was used to isolate DNA:RNA hybrids and by extension R-loops from nuclear extracts to identify the associated R-loop proteome^22^. In the second method, a synthetic DNA:RNA hybrid was used as a bait to enrich for factors that bind hybrid nucleic acids^23^. Both S9.6 and Hybrid immunoprecipitation (that we term S9.6 IP and Hybrid IP, respectively) share some common and other unique drawbacks. The conditions of immunoprecipitation using both these methods allow for recovery of stable R-loop interactors, but transient and weakly bound interactors that are sensitive to high salt and detergent washes are likely to be lost. Neither method is amenable to use with denaturing conditions. In addition, the Hybrid IP technique cannot enrich for proteins that bind the ssDNA component of R-loops.

The identification of transient interactions has been facilitated in recent years by several proximity-based labeling approaches^24–27^. The unifying theme in these diverse technologies is the transfer of a biotin label from the target to proximate proteins (and RNA in the case of IPL and APEX), that can be purified by streptavidin affinity and identified by mass spectrometry. Based on the established function of RNase H in R-loop regulation, we used TurboID^25^ to uncover the RNase H proximal proteome that we propose may also identify factors that function at R-loop structures. We identify homeodomain and zinc finger containing proteins as highly enriched in proximity to RNase H. Furthermore, we identify the activity-dependent neuroprotector homeodomain protein (ADNP), one of the most frequently mutated and high-confidence autism spectrum genes^28,29^, and show that it directly regulates R-loop structures.

## Results

**Fig. 1:**
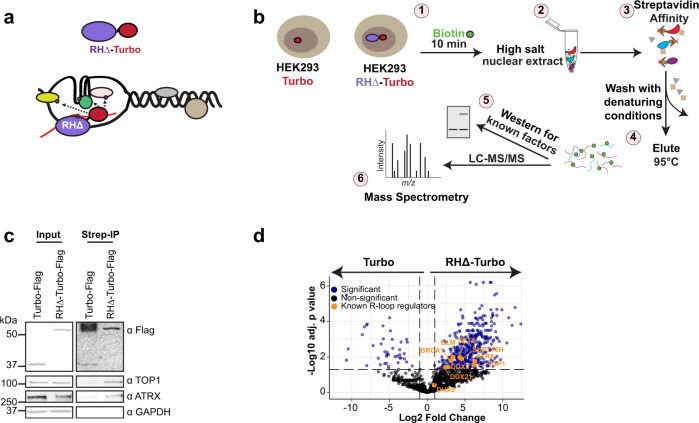
TurboID identifies the RNase H proximal proteome. **a** Schematic showing RHΔ-Turbo localizing to R-loops and biotinylating proximal proteins. **b** Schematic of TurboID. Stable HEK293 cell lines expressing RHΔ-Turbo or Turbo alone were pulsed with biotin for 10 min (1). High salt nuclear extract was prepared (2), and biotinylated proteins isolated by streptavidin affinity purification (3). Beads were washed with denaturing conditions to minimize non-specific interactions and bound biotinylated proteins were eluted (4), tested for the presence of known R-loop regulators by western blot (5), and subjected to mass spectrometry (6). **c** Western blot for TOP1, ATRX, and GAPDH in HEK293 Turbo and RHΔ-Turbo input (left) and in streptavidin pull-downs (right). Antibodies are indicated on the right. Turbo and RHΔ-Turbo are detected with anti-Flag antibody. **d** Volcano plot showing log2 fold changes in protein intensities on the x-axis and −log10 adjusted *p*-values (Student’s two-sided *t*-test with Benjamini–Hochberg adjustment for multiple comparisons) on the y-axis. Significantly enriched proteins (blue, *p* < 0.05) and non-significant in black. Known R-loop regulators (orange) are labeled. Source data underlying (**c**) are provided as a Source data file.

To identify factors with potential to function at R-loops in vivo through their proximity to RNase H, we used TurboID^25^, a proximity labeling method that leverages the promiscuous activity of an engineered biotin ligase with enhanced catalytic activity compared to the *E. coli* biotin ligase used in BioID^27^. We fused biotin ligase to a catalytically inactive RNase H (RHΔ-Turbo) (Fig. 1a) that can bind but cannot resolve DNA:RNA hybrids, and expressed the fusion protein in HEK293 (Supplementary Fig. 1a). In TurboID, the biotinylation reaction is initiated by the addition of exogenous biotin to the culture media. We optimized biotinylation time by treating cells with biotin for various lengths of time (Supplementary Fig. 1b). Nuclear extracts isolated prior to addition of biotin contained very few, if any, biotinylated proteins. Upon addition of biotin, we observed significant increase in biotin signal even at the shortest time point of 10 min (Supplementary Fig. 1b). After treatment with biotin (Fig. 1b, step 1), high salt nuclear extracts were prepared (Fig. 1b, step 2) and passed through a streptavidin affinity column (Fig. 1b, step 3). Biotinylated proteins that bound streptavidin were washed with very stringent conditions to minimize enrichment of proteins that non-specifically interact with the streptavidin affinity resin. Bound proteins were eluted (Fig. 1b, step 4) and analyzed by Western blot to confirm the presence of known R-loop regulators (Fig. 1b, step 5). Eluates were then processed for mass spectrometry (Fig. 1b, Step 6). As expected, both Turbo-F and RHΔ-Turbo-F were recovered from streptavidin beads (Fig. 1c); these proteins are likely self-biotinylated by the Turbo moiety. In addition, TOP1 and ATRX, proteins with known functions at R-loops^15,17^, were enriched in the RHΔ-Turbo samples but not in Turbo alone (Fig. 1c). In contrast, a control protein unrelated to R-loops, GAPDH, was not biotinylated in either sample. Silver staining confirmed that RHΔ-Turbo samples contained a different protein content compared to Turbo alone (Supplementary Fig. 1c). We performed RHΔ-TurboID in 3 biological replicates (Fig. 1b, step 6) and identified 441 total proteins that were significantly enriched (adjusted *p*-value<0.05, log2 fold change >1) in the RHΔ-Turbo sample over Turbo alone (Fig. 1d, Supplementary Data 1). These proteins included a number of known R-loop regulators^14,15,30–33^ (Fig. 1d, orange labels), and many factors with potential to function at R-loops.

### ATRX RNA binding activity inhibits R-loop formation

**Fig. 2:**
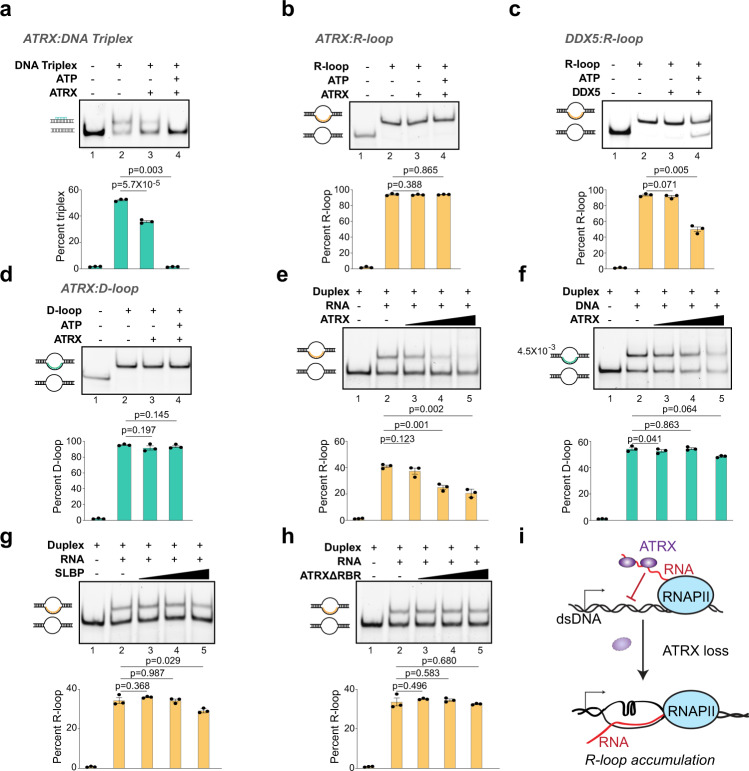
ATRX RNA binding activity inhibits R-loop formation. **a** DNA strand displacement assay with full-length ATRX (25 nM) and 1 nM DNA triplex substrates with or without ATP as indicated. Positions of duplex and DNA triplex are shown. For all subsequent panels in this figure, quantification of 3 independent experiments is shown as mean values ± SEM. p, two-sided Student’s t-test. **b** R-loop resolution assay with full-length ATRX (25 nM) and 1 nM R-loop substrates with or without ATP as indicated. Positions of duplex and R-loops are shown. **c** R-loop resolution assay with 25 nM DDX5 and 1 nM R-loop substrates without or with ATP as indicated. **d** D-loop resolution assay with full-length ATRX (25 nM) and 1 nM D-loop substrates with or without ATP as indicated. Positions of duplex and D-loops are shown. **e** R-loop formation assay with 1.25 nM DNA duplex, 3.75 nM RNA and increasing concentrations of full-length ATRX (5, 25, 125 nM). **f** D-loop formation assay 1.25 nM DNA duplex, 3.75 nM ssDNA and increasing concentrations of full-length ATRX (5, 25, 125 nM). **g** R-loop formation assay with 1.25 nM DNA duplex, 3.75 nM RNA, and increasing concentrations of full-length SLBP (5, 25, 125 nM). **h** R-loop formation assay with 1.25 nM DNA duplex, 3.75 nM RNA, and increasing concentrations of ATRXΔRBR (5, 25, 125 nM). **i** Model: ATRX interacts with repeat containing RNAs through its RNA binding region and prevents their incorporation into R-loops. ATRX deletion allows RNAs to hybridize to their complementary DNA, resulting in R-loop stabilization and accumulation. Source data underlying (**a**–**h**) are provided as a Source data file.

We identified ATRX by RHΔ-TurboID (Fig. 1d). While ATRX loss is associated with increase in R-loops at telomeres^15^ and repeat instability^34^, the mechanism by which it suppresses R-loops is not clear. ATRX can bind G quadruplexes (G4)^35^ that frequently occur on the non-template strand of R-loops because of high GC skew. However, ATRX cannot resolve these structures in vitro^36^, suggesting another mechanism for R-loop suppression. Several studies show that ATRX can displace a third strand of DNA from DNA triplex structures^37,38^. DNA triplexes form when the third DNA strand occupies the major groove of the double helix and forms Hoogsteen (or reverse Hoogsteen) hydrogen bonds with the purines in the Watson-Crick strands^39^. In contrast, R- and D-loops form when the third strand of RNA or DNA, respectively, invades dsDNA to form Watson-Crick base pairs with the template strand, resulting in extrusion of the non-template strand^4^. Whether ATRX can resolve R-loops has not been tested. We purified full-length ATRX (Supplementary Fig. 2) and confirmed that it was enzymatically active by assaying its ability to resolve DNA triplexes in vitro (Fig. 2a). While addition of ATRX resulted in a slight destabilization of DNA triplexes even in the absence of ATP (Fig. 2a, compare lanes 2 and 3), addition of ATP resulted in almost complete resolution of DNA triplex substrates (Fig. 2a, compare lanes 2 and 4). Therefore, as reported previously, ATRX is able to displace the third DNA strand from dsDNA in the context of DNA triplexes in an ATP-dependent manner.

Next, we examined whether ATRX can resolve R-loops in vitro. We found that addition of ATRX, without or with ATP, at concentrations at which it can efficiently resolve DNA triplexes (Fig. 2a), does not result in any change in R-loop integrity (Fig. 2b, compare lanes 2, 3, and 4). However, in identical experimental conditions, R-loop substrates were efficiently resolved by DDX5 (Fig. 2c, Supplementary Fig. 2), an RNA helicase with well-characterized ability to disrupt R-loops^14^ and that was also identified by RHΔ-TurboID (Fig. 1d). To determine if ATRX acts only on triplex nucleic acid structures with a DNA third strand, we tested whether ATRX is able to resolve D-loops in vitro. Similar to R-loops, ATRX is also unable to resolve D-loops (Fig. 2d, compare lanes 2, 3, and 4). This suggests that while ATRX is able to disrupt the Hoogsteen hydrogen bonds formed in DNA triplexes, it is unable to act on the Watson-Crick base pairs formed in R- and D-loops. Thus, we conclude that under the experimental conditions where both DDX5 (Fig. 2c) and ADNP (see below) can resolve R-loops, full-length ATRX is unable to resolve R-loops.

ATRX is a high-affinity RNA binding protein^40,41^. We asked whether its binding to RNA would inhibit the formation of R-loops (Fig. 2e). We incubated the RNA strand with increasing concentrations of full-length ATRX and added this mixture to DNA duplex in an R-loop assembly reaction. Resolution of the products on a native gel showed that R-loops form in the absence of ATRX (Fig. 2e, lane 2). The extent of R-loop formation diminishes with increasing concentrations of ATRX (Fig. 2e, compare lanes 3–5). ATRX binds dsDNA but shows significantly reduced affinity for ssDNA^40^. We tested whether incubation of ATRX with the DNA third strand is able to inhibit D-loop formation. We found that D-loops form in the absence of ATRX (Fig. 2f, lane 2) and that ATRX presence does not significantly inhibit D-loop formation (Fig. 2f, lanes 3–5). To determine if R-loop inhibition is a property of other RNA binding proteins, we examined whether the stem-loop binding protein (SLBP)^42^ inhibits R-loop formation (Fig. 2g, Supplementary Fig. 2). Our results indicate that SLBP only inhibited R-loop assembly at the highest concentration (Fig. 2g, lane 5). Finally, we asked whether the RNA binding property of ATRX was responsible for its ability to inhibit R-loops. We recently identified the ATRX RNA binding region (ATRX-RBR) and found that its deletion (ATRXΔRBR) resulted in severely reduced interactions with its cognate RNAs in vitro^43^. We found that ATRXΔRBR has no measurable effect on R-loop formation even at the highest concentration (Fig. 2h, compare lanes 2–5, Supplementary Fig. 2). Thus, we conclude that ATRX RNA binding inhibits the formation of R-loops in vitro. We propose that ATRX interactions with its cognate RNAs in vivo prevent the RNA from interacting with the template DNA strand and suppress R-loops (Fig. 2i). When ATRX expression is reduced or lost, RNAs can then pair with DNA to result in R-loop accumulation.

### Proximity labeling identifies homeodomain and zinc finger proteins at R-loops in vivo

**Fig. 3:**
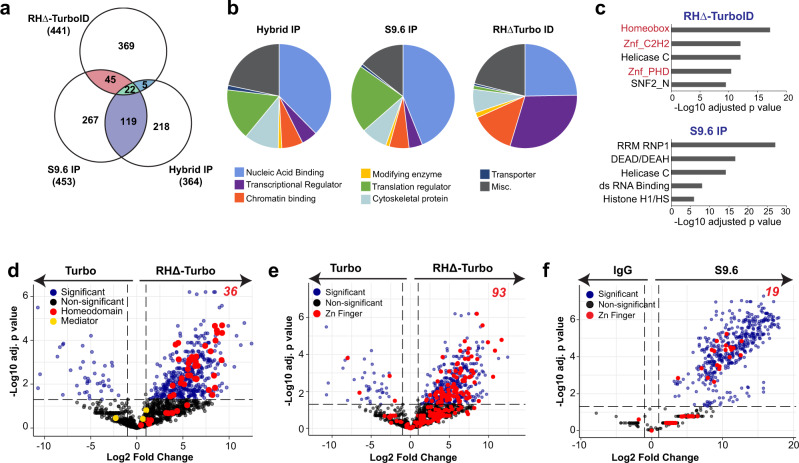
Homeodomain and zinc finger proteins are enriched at R-loops. **a** Venn diagram showing overlap between enriched proteins in RHΔ-TurboID, S9.6 IP, and DNA:RNA hybrid IP. Total number of significantly enriched proteins (adjusted *p* value < 0.05), and the numbers shared between the three methods are indicated. **b** Distribution of R-loop interactors from RHΔ-TurboID, S9.6 IP, and DNA:RNA hybrid IP based on molecular functions. **c** Top 5 most significantly enriched domains in RHΔ-TurboID and S9.6 IP. Adjusted *p*-values calculated by Enrichr using hypergeometric test with Benjamini–Hochberg adjustment for multiple comparisons. **d** Volcano plot showing log2 fold changes in protein intensities on the x-axis and −log10 adjusted *p*-values (Student’s two-sided *t*-test with Benjamini–Hochberg adjustment for multiple comparisons) on the y-axis in RHΔ-TurboID. Significantly enriched proteins in blue (*p* < 0.05) and non-significant in black. Homeodomain proteins are highlighted in red; components of the Mediator complex are highlighted in yellow. **e** Volcano plot showing log2 fold changes in protein intensities on the x-axis and −log10 adjusted *p*-values (Student’s two-sided *t*-test with Benjamini–Hochberg adjustment for multiple comparisons) on the y-axis in RHΔ-TurboID. Significantly enriched proteins (blue, *p* < 0.05) and non-significant in black. Zinc finger containing proteins are highlighted in red. **f** Volcano plot showing log2 fold changes in protein intensities on the x-axis and −log10 adjusted *p*-values (Student’s two-sided *t*-test with Benjamini–Hochberg adjustment for multiple comparisons) on the y-axis in S9.6 IP. Significantly enriched proteins (blue, *p* < 0.05) and non-significant in black. Zinc finger containing proteins are highlighted in red.

We asked how RHΔ-TurboID compared to two other in vitro approaches, S9.6 IP^22^ and Hybrid IP^23^, that have previously identified R-loop interactors (Supplementary Data 1). These methods are based on the co-immunoprecipitation of proteins associated with R-loops, as opposed to our in vivo proximity labeling approach. We identified 67 shared proteins between RHΔ-TurboID and S9.6 IP and 27 proteins between RHΔ-TurboID and Hybrid IP. Only 22 proteins, including DDX5, a well-characterized R-loop regulator^14^, were shared between all three datasets (Fig. 3a). We found that both S9.6 IP and hybrid IP recovered a large number of proteins involved in translation compared to RHΔ-TurboID (Fig. 3b, Supplementary Data 1). Our analyses of the S9.6 IP dataset showed that ribosomal proteins showed the highest fold change and comprised almost 17% of all significantly enriched proteins (77 out of 453, Supplementary Figs. 3a, 3b). In contrast, RHΔ-TurboID did not show an enrichment for ribosomal proteins (4 out of 441, Supplementary Fig. 3b, c) and was more sensitive in detecting lowly expressed proteins as demonstrated by the large proportion of transcriptional regulators (Fig. 3b). We performed a domain enrichment analysis^44,45^ to identify specific protein domains that were enriched in RHΔ-TurboID and S9.6 IP (Fig. 3c, Supplementary Data 1). Our results showed that both RHΔ-TurboID and S9.6 IP contained proteins with helicase-related domains (Helicase C, SNF2_N, DEAD/DEAH) that are expected to have functions at R-loops. Interestingly, RHΔ-TurboID was characterized by an enrichment of “homeobox” and “zinc fingers” domains. Both homeodomain and zinc finger proteins were absent in S9.6 IP (Fig. 3c). To ascertain why homeodomain and zinc finger proteins were only recovered by RHΔ-TurboID, we compared the abundance of R-loop interactors identified by RHΔ-TurboID and S9.6 IP to their abundance in the proteome (Supplementary Fig. 3d). RHΔ-TurboID and S9.6 were compared to the HEK293 and HeLa proteomes, respectively^46^. The distribution of the RHΔ-TurboID dataset (Supplementary Fig. 3d, orange) appeared uniform while proteins identified by S9.6 IP (Supplementary Fig. 3d, teal) clearly separated into two distinct populations: one that overlapped with RHΔ-TurboID, and a second that corresponded to proteins that are very abundant in the HeLa proteome. Thus, the enrichment of homeodomain and zinc finger proteins is likely because, compared to S9.6 IP, RHΔ-TurboID can detect less abundant proteins (Supplementary Fig. 3d, left half of orange contour plot) including many transcriptional regulators that may have potential function at R-loops.

R-loops are co-transcriptional structures that typically form near the 5′ end of genes^5^, where transcription factors with homeodomain and zinc fingers are likely to localize^47,48^. RHΔ-TurboID identified 36 homeodomain (Fig. 3d) and 93 zinc finger (Fig. 3e) proteins. S9.6 IP did not recover any homeodomain proteins and only identified 19 zinc finger proteins (Fig. 3f). The abundance of homeodomain and zinc finger containing proteins in RHΔ-TurboID raises the possibility that their enrichment may be because of their general proximity to transcription events as opposed to a direct function at R-loops. To distinguish between these, we looked at the enrichment of components of the Mediator complex, an abundant multi-subunit transcription-associated protein complex^49^. Interestingly, we did not obtain any significantly enriched peptides from the ~30 subunit mediator complex by RHΔ-TurboID (Fig. 3d, yellow), indicating that the identification of homeodomain and zinc finger proteins by RHΔ-TurboID was due to their specific enrichment in the proximity of R-loops. An overlap of enriched homeodomain and zinc finger proteins identified 5 proteins that contained both protein domains. Because ADNP, which contains 9 zinc fingers and a homeodomain, showed the highest peptide abundance across three replicates (Supplementary Fig. 3e), is enriched in RHΔ-Turbo compared to Turbo alone (Supplementary Fig. 3f), and is relevant to autism spectrum disorders, we chose to further examine its function at R-loops.

### ADNP resolves R-loops in vitro

**Fig. 4:**
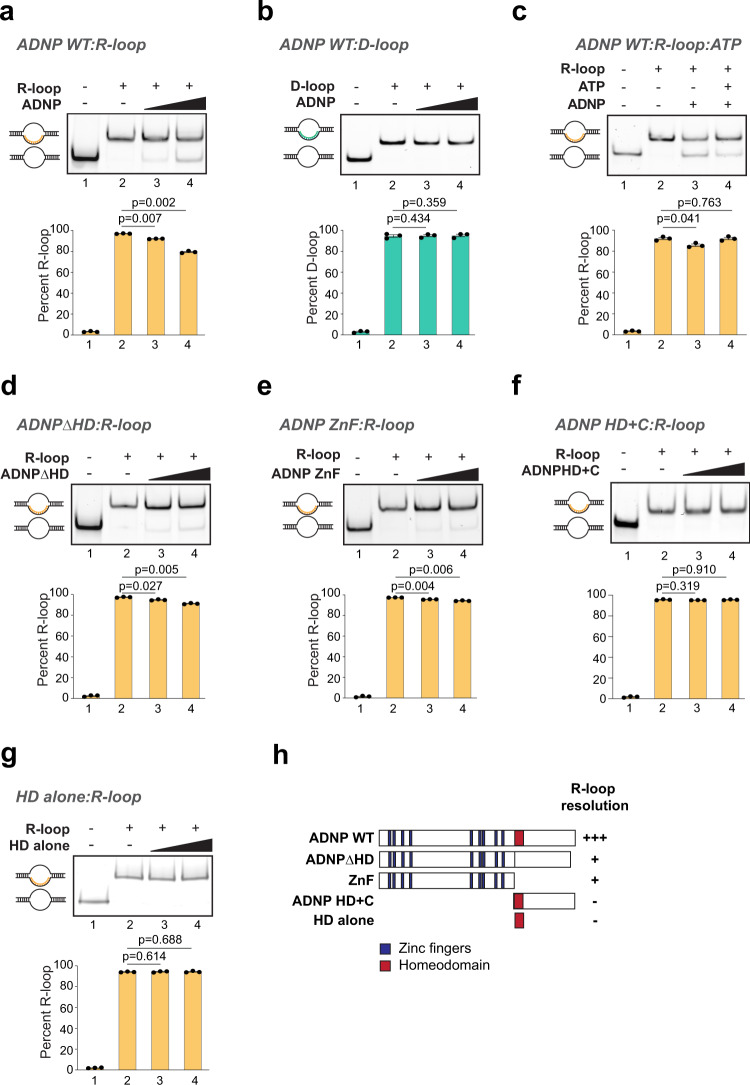
ADNP resolves R-loop structures in vitro. **a** R-loop resolution assay with 40 nM and 200 nM ADNP WT and 1 nM R-loop substrates. Positions of duplex and R-loops are shown. For all resolution assays, quantification of 3 independent experiments is shown as mean values ± SEM. Two-sided Student’s *t* test *p*-values are shown. **b** D-loop resolution assay with 40 nM and 200 nM ADNP WT and 1 nM D-loop substrates. Positions of duplex and D-loops are shown. **c** R-loop resolution assay with 200 nM ADNP WT and 1 nM R-loop substrates without or with ATP as indicated. **d** R-loop resolution assay with 40 nM and 200 nM ADNPΔHD and 1 nM R-loop substrates. **e** R-loop resolution assay with 40 nM and 200 nM ADNP ZnF and 1 nM R-loop substrates. **f** R-loop resolution assay with 40 nM and 200 nM ADNP homeodomain and C terminus and 1 nM R-loop substrates. **g** R-loop resolution assay with 40 nM and 200 nM ADNP homeodomain alone and 1 nM R-loop substrates. **h** Summary of R-loop resolution activities of full-length ADNP WT, ADNPΔHD, ADNP ZnF, ADNP HD + C, and the homeodomain alone. Source data underlying (**a**–**g**) are provided as a Source data file.

ADNP is a homeodomain protein that contains 9 zinc fingers and a homeodomain^50^. Both homeodomains and zinc fingers bind nucleic acids. To gain a molecular understanding of how ADNP functions at R-loops, we first tested its ability to interact with R-loops in vitro. We performed EMSA using full-length human ADNP (ADNP WT) expressed and purified from Sf9 insect cells (Supplementary Fig. 4a) and reconstituted R-loops. Surprisingly, upon addition of ADNP protein to reconstituted R-loops, instead of a robust mobility shift that would indicate binding, we observed a consistent ADNP concentration-dependent resolution of the R-loop substrates (Fig. 4a, Supplementary Fig. 4b). In a similar experiment, ADNP is unable to resolve D-loop structures (Fig. 4b). To confirm that our ADNP protein sample did not contain a contaminating ribonuclease, we incubated ADNP with the RNA used to generate R-loop substrates and found that the RNA strand remained intact and was not degraded (Supplementary Fig. 4c). ADNP does not have annotated helicase or ATPase domains. We reasoned that if R-loop resolution occurs because of the presence of a contaminating ATP-dependent helicase, resolution activity would be stimulated by the addition of ATP. Interestingly, we found that the ability of ADNP to resolve R-loops is independent of ATP hydrolysis, and is instead slightly inhibited by ATP (Fig. 4c, compare lanes 3 and 4). This may occur if ADNP binding to ATP prevents its association with R-loops to facilitate their resolution. Finally, to discount that R-loop resolution activity resulted from a protein contaminant that associated with ADNP in Sf9 cells, we purified full-length hADNP from a bacterial expression system. Expression of ADNP in bacteria generates many truncation products and results in the recovery of low levels of full-length ADNP (Supplementary Fig. 4d). Nevertheless, purified full-length ADNP from bacteria is also able to resolve R-loops in vitro (Supplementary Fig. 4e). Together, our results provide evidence that ADNP resolves R-loops by an ATP-independent mechanism distinct from those reported for other R-loop resolving helicases.

To identify the domain of ADNP that confers R-loop resolution function, we expressed and purified ADNP mutants that lacked the homeodomain (ADNPΔHD), or that contained only the zinc fingers (ADNP ZnF), the homeodomain and C terminus (HD + C), or the homeodomain (HD alone) (Supplementary Fig. 4a). At the same concentrations, ADNPΔHD and ADNP ZnF displayed R-loop resolving activity in vitro (Fig. 4d, e), albeit lower than the WT protein (compare to Fig. 4a). ADNP fragment containing the homeodomain and the C terminus (HD + C) or the homeodomain alone were unable to resolve R-loops (Fig. 4f, g). We conclude that the R-loop resolution ability of ADNP is contained within the zinc fingers (Fig. 4h) and that the homeodomain is necessary but not sufficient for maximum R-loop resolution by ADNP.

### ADNP suppresses R-loop formation at its binding sites genome-wide

To elucidate ADNP function at R-loops in vivo, we examined R-loop levels in ADNP knock-out (KO) mouse embryonic stem cells (mESCs). We generated ADNP KO mESCs with CRISPR/Cas9 by deleting the coding sequence for all 9 zinc fingers and the homeodomain using two guide RNAs (Supplementary Fig. 5a, top). We obtained several ADNP KO clones that showed no detectable levels of ADNP protein (Supplementary Fig. 5a, bottom). We also generated ADNP HA-V5 knock-in mESCs (ADNP-KI, Supplementary Fig. 5b). First, to determine if R-loop levels were globally increased in ADNP KO, as we would expect if ADNP functioned as an R-loop suppressor genome-wide, we performed dot blot analysis using the S9.6 antibody that recognizes DNA:RNA hybrids (Supplementary Fig. 5c, top). Antibodies that recognize dsDNA were used as a loading control (Supplementary Fig. 5c, bottom). S9.6 signal was not visibly higher in ADNP KO clones compared to parental controls. Thus, we conclude that ADNP loss does not result in widespread R-loop increases in vivo.

**Fig. 5:**
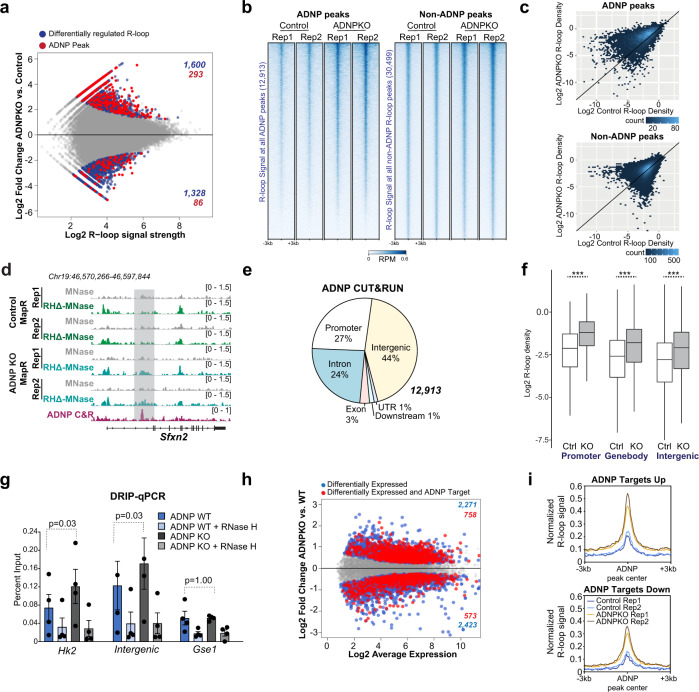
ADNP suppresses R-loops at its binding sites in vivo. **a** MA plot of MapR signal in 61,652 R-loop peaks between Control (ADNP-KI) and ADNP KO mESCs. Blue dots indicate 2928 significant differential R-loop sites (FDR < = 0.05, DiffBind; 1600 up and 1328 down in KO). Significant differential R-loops that overlap an ADNP CUT&RUN peak (293 up, 86 down) are colored in red. **b** Heatmap of normalized MapR signal across 6-kb windows centered on ADNP peak locations (left) or control R-loop peaks that do not overlap ADNP peaks (right). Rows are sorted in decreasing order by mean signal across all samples. **I** Hexagonal-binned scatterplot of mean normalized MapR signal in control and ADNP KO mESCs across the regions shown in (**b**). **d** Genome browser view of the *Sfxn2* gene showing MapR signal (RPM) in control and ADNP KO mESCs and ADNP CUT&RUN signal (RPM) in control mESCI. **e** Pie chart displaying distribution of 12,913 ADNP peaks across genomic features. **f** Boxplot displaying log2 normalized R-loop read densities in control and ADNP KO mESCs across ADNP peaks, grouped by genomic feature. ***p < 2.2 × 10^−16^ (Welch’s two-sided *t*-test; *n* = 3432 promoter peaks, 3653 gene body peaks, or 5828 intergenic peaks). Box, 25th percentile – median – 75th percentile. Whiskers extend to 1.5x interquartile range; outliers not displayed. **g** DRIP-qPCR results using the S9.6 antibody in WT and ADNP KO mESCs at an *Hk2* gene region, an intergenic region, and a *Gse1* gene control region. Bar chart, mean ± SEM; individual values shown as Dots. *p*, Welch’s two-sided *t*-test (*n* = 4 biologically independent samples). **h** MA plot of RNA-Seq expression for 16,195 genes between WT and ADNP KO mESCs. Blue dots indicate differentially expressed genes (adjusted *p*-value <=0.05; *p*-values computed by edgeR with Benjamini–Hochberg adjustment for multiple comparisons). Red dots indicate differentially expressed genes with an ADNP CUT&RUN peak within 3 kb of the gene body. **i** Signal plot of normalized control and ADNP KO MapR signal over ADNP peaks associated with ADNP target genes that are upregulated (1219 peaks) or downregulated (1040 peaks) in ADNP KO mESCs relative to WT. Source data underlying (**g**) are provided as a Source data file.

We assayed the genomic distribution of R-loops in ADNP-KI, which is called ‘control’ in all subsequent experiments, and ADNP KO mESCs using MapR, a sensitive, antibody independent technique that utilizes RNase H-guided micrococcal nuclease (RHΔ-MNase) to cleave and release R-loops for sequencing^51,52^. Principal component analysis showed that control and ADNP KO R-loop replicates clustered separately (Supplementary Fig. 5d). Comparison of R-loops between control and ADNP KO identified 2928 differentially regulated R-loops (out of 61,652 total), with 1600 increased and 1328 decreased upon ADNP KO (Fig. 5a, blue dots). To facilitate identification of direct versus indirect effects of ADNP on R-loops, we performed ADNP CUT&RUN^53,54^ to identify ADNP binding sites. We generated an ADNP antibody against a C-terminal fragment of human ADNP that is highly conserved between mouse and human (Supplementary Fig. 5e). Western blot detected ADNP signal at the expected molecular weight (~150 kDa) in WT mESCS, but not in ADNP KO mESCs (Supplementary Fig. 5f), confirming that our ADNP antibody specifically detects ADNP protein. Previous studies show that ADNP is nuclear and shows enrichment at pericentromeres in mouse embryonic fibroblasts^55^. Immunostaining of mESCs with ADNP antibody showed localization to DAPI dense nuclear foci that correspond to pericentromeres (Supplementary Fig. 5g, left) while no signal was detected in ADNP KO mESCs (Supplementary Fig. 5g, right), further confirming antibody specificity. ADNP CUT&RUN identified a total of 12,913 ADNP peaks in control (ADNP KI) mESCs. We determined overlap of all mESC R-loops with these ADNP CUT&RUN peaks and found that of 61,652 R-loops, 7506 (12.2%) overlapped an ADNP binding site. Next, we examined differentially regulated R-loops and found that 293 of the 1600 R-loops that were significantly increased in ADNP KO (18.3%) overlapped an ADNP peak, while only 86 of 1328 lost R-loops (6.48%) contained an ADNP site (Fig. 5a, red dots). This represented a 1.5-fold over-enrichment of ADNP binding sites in gained R-loops, compared to a 1.88-fold under-enrichment in lost R-loops (*p* = 4.7 × 10^−13^, 2.6 × 10^−12^, respectively; hypergeometric test).

Next, we examined R-loop signal at ADNP binding sites in control and ADNP KO mESCs and found that R-loop levels were increased across the majority of ADNP sites (Fig. 5b, c), with good correlation across biological replicates (Pearson correlation coefficient = 0.75, Supplementary Fig. 5h). In contrast, R-loop levels between control and ADNP KO are relatively unchanged at R-loop peaks that do not overlap ADNP sites (Fig. 5b, c), consistent with our observation that ADNP loss does not result in genome-wide R-loop increase. At ADNP binding sites within the *Sfxn2* and *Vps36* genes, ADNP KO mESCs show notable R-loop increase compared to control (Fig. 5d, Supplementary Fig. 5i). *Sfxn2* and *Vps36* are expressed and contain R-loops in proximity to the TSS that do not show ADNP enrichment and that do not change between control and ADNP KO (Fig. 5d, Supplementary Fig. 5i), again strengthening the notion that ADNP loss affects R-loops specifically at its own binding sites. ADNP localizes to both genic and intergenic sites across the genome (Fig. 5e). We found that R-loops show significant increase in ADNP KO across all feature types: at promoters, within genes, and at intergenic regions that contain ADNP binding sites (Fig. 5f).

To further validate our findings using an independent approach, we performed DNA-RNA immunoprecipitation (DRIP)^5^ and analyzed 2 candidate loci, one within the *Hk2* gene and another at an intergenic site, that show R-loop increase in ADNP KO by MapR (Supplementary Fig. 5j). DRIP-qPCR shows that both regions have low R-loop signal in WT mESCs that increase in ADNP KO (Fig. 5g). Treatment of samples with RNase H prior to S9.6 immunoprecipitation resulted in signal decrease in both WT and ADNP KO, attesting to the presence of bona fide R-loops. A control locus within the *Gse1* gene that does not show R-loop change by MapR (Supplementary Fig. 5j) is unchanged in ADNP KO by DRIP-qPCR (Fig. 5g).

R-loops form as a consequence of active transcription^5,51,56^ and ADNP functions as a transcriptional repressor^57^. Therefore, the R-loop accumulation we observe in ADNP KO mESCs could simply reflect increased transcription from ADNP target genes upon ADNP loss. To exclude this possibility, we performed RNA-Seq on WT and ADNP KO mESCs and identified 4694 out of 12,351 detectable genes as differentially expressed between WT and ADNP KO (adjusted *p*-value < =0.05, Fig. 5h, Supplementary Data 2). Next, we defined “ADNP targets” as genes containing an ADNP peak in the gene body or within 3 kb upstream or downstream; 3322 expressed genes (26.9%) met this criterion. 758 ADNP targets were upregulated and 573 were downregulated in ADNP KO compared to WT. This represented a significant enrichment of ADNP targets over background (*p* = 2.1 × 10^−14^, hypergeometric test) in upregulated genes (Supplementary Fig. 5k), consistent with ADNP’s role as a transcription repressor, and a significant under-enrichment (*p* = 2.7 × 10^−5^, hypergeometric test) in downregulated genes (Supplementary Fig. 5k). We then investigated R-loop signal at ADNP targets and found R-loop signal increased at ADNP binding sites associated with upregulated genes (Fig. 5i). Notably, R-loop signal also increased at ADNP sites associated with downregulated genes (Fig. 5i), suggesting these R-loop gains at ADNP sites are not coupled to transcriptional changes, but are instead a direct consequence of loss of ADNP binding. Thus, our data demonstrate an R-loop suppression function for ADNP at its own binding sites.

### ADNP homeodomain deletion results in R-loop accumulation and compromises neuronal differentiation

**Fig. 6:**
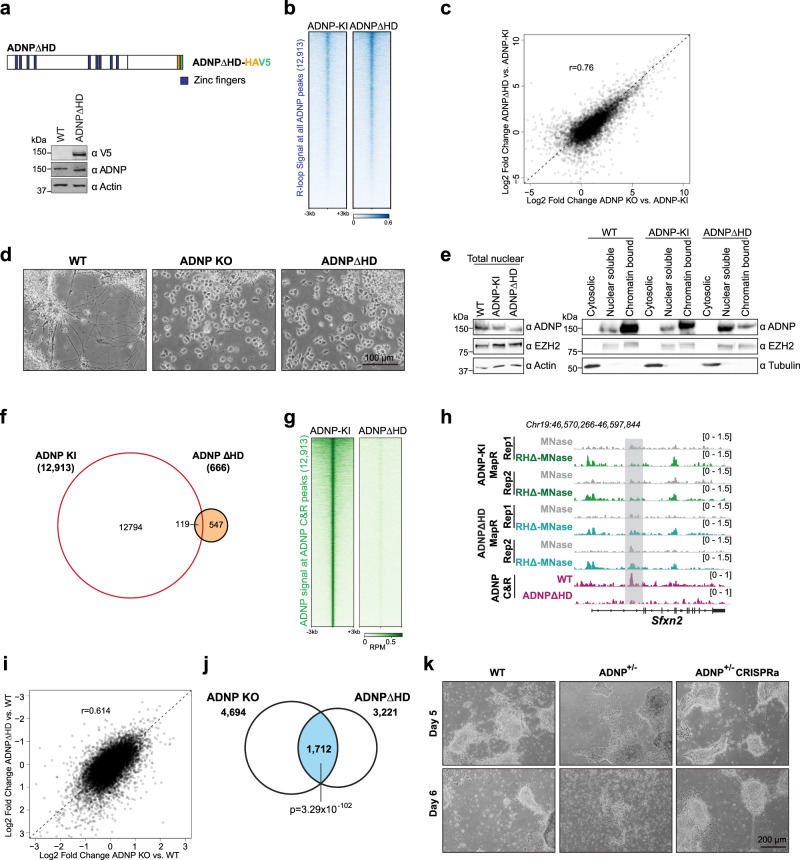
ADNP homeodomain deletion results in protein mislocalization and R-loop accumulation. **a** Schematic of HA and V5 epitope-tagged ADNPΔHD (top). Zinc fingers are in blue. Western blot for V5 tag and ADNP in parental WT and ADNPΔHD mESCs (bottom). Actin serves as loading control. Antibodies indicated on the right. **b** Heatmap of normalized MapR signal in ADNP-KI and ADNPΔHD mESCs across 6-kb windows centered on ADNP CUT&RUN peaks. Rows are sorted in decreasing order by mean signal across all samples. **c** Scatterplot of log2 fold changes in R-loop read density between control and ADNP KO mESCs and log2 fold changes between ADNP-KI and ADNPΔHD mESCs across 12,913 ADNP peaks. Pearson correlation, 0.76. **d** Representative images of WT, ADNP KO, and ADNPΔHD cells on day 5 of differentiation. **e** Western blot for ADNP, EZH2, Actin, and Tubulin in WT, ADNP-KI, and ADNPΔHD mESCs total nuclear extract (left), and upon fractionation into cytosolic, nuclear soluble and chromatin-bound fractions (right). Antibodies are indicated on the right. **f** Venn diagram showing overlap between 12,913 ADNP CUT&RUN peaks and 666 ADNP CUT&RUN peaks called in ADNP-KI and ADNPΔHD, respectively. **g** Heatmap of ADNP CUT&RUN signal (RPM) in ADNP-KI and ADNPΔHD mESCs across 6-kb windows centered on 12,913 ADNP peaks. Rows are sorted in decreasing order by mean signal across all samples. **h** Genome browser view of the *Sfxn2* gene showing MapR signal (RPM) in ADNP-KI and ADNPΔHD mESCs and ADNP CUT&RUN signal (RPM) in ADNP-KI and ADNPΔHD mESCs. **i** Scatterplot of log2 fold changes in RNA-Seq expression between WT and ADNP KO mESCs and log2 fold changes between WT and ADNPΔHD mESCs for 11,973 expressed genes. Pearson correlation, 0.614. **j** Venn diagram showing overlap between 4694 genes differentially expressed in ADNP KO and 3221 genes differentially expressed in ADNPΔHD. *p*-value of overlap = 3.29 × 10^−102^, hypergeometric test. **k** Representative images of WT, ADNP^+/−^, and ADNP^+/−^ CRISPRa cells on days 5 and 6 of neuronal differentiation. Source data underlying **a**, **d**, **e**, **k** are provided as a Source data file.

Our data indicate that deletion of the homeodomain significantly affects the R-loop resolution activity of ADNP (Fig. 4d) and that ADNP loss results in R-loop accumulation at its binding sites (Fig. 5). To determine whether loss of the ADNP homeodomain is sufficient to cause R-loop accumulation in vivo, we engineered mESCs to exclusively express ADNP lacking the homeodomain (ADNPΔHD) (Fig. 6a). We simultaneously inserted HA and V5 epitope tags as with our ADNP knock-in mESCs (Supplementary Fig. 5b). We confirmed that ADNP levels in ADNPΔHD were similar to parental mESCs (Fig. 6a). We evaluated R-loop levels at ADNP sites in ADNPΔHD by MapR and found that, as with ADNP KO cells, R-loops were increased across ADNP binding sites in ADNPΔHD compared to control (ADNP-KI) (Fig. 6b). We compared our ADNP KO and ADNPΔHD R-loop datasets and found that R-loop increases compared to knock-in control were similar across the majority of ADNP sites (Pearson correlation = 0.76, Fig. 6c). Principal component analysis of MapR data across ADNP sites revealed that ADNP KO and ADNPΔHD R-loops clustered closer together and further apart from control (ADNP-KI) R-loops (Supplementary Fig. 6a). These findings indicate that the ADNP homeodomain is required for ADNP-mediated R-loop suppression in vivo and that homeodomain deletion has comparable effects on R-loop regulation as total ADNP loss.

Previous studies have established that ADNP plays a critical role in neural differentiation^57^. ADNP KO mESCs cannot differentiate into neural progenitor cells (NPCs) or neurons^57–59^. In ADNP syndrome, recurring nonsense mutations result in protein truncation before the homeodomain^28,60^. To determine if the homeodomain of ADNP contributes to proper neuronal differentiation, we differentiated mESCs toward the neuroectoderm lineage by withdrawal of leukemia inhibitory factor (LIF) and addition of growth factors including basic fibroblast growth factor (bFGF) and smoothened agonist (SAG)^61,62^. In the undifferentiated state, WT, ADNP KO, and ADNPΔHD mESCs appear indistinguishable and express similar levels of pluripotency markers including Oct4, Nanog, and Sox2 (Supplementary Fig. 6b). Upon differentiation, WT mESCs form long extensions on day 5 that morphologically resemble neurites (Fig. 6d, left). WT NPCs properly downregulate expression of pluripotency markers while upregulating neural lineage markers (Supplementary Fig. 6c). As reported previously^57,58^, ADNP KO mESCs showed significant cell death upon induction of differentiation and failed to form NPCs (Fig. 6d, middle). Interestingly, the ADNPΔHD differentiation phenotype closely resembled ADNP KO, with increased cell death upon differentiation and a failure to form NPCs by day 5 (Fig. 6d, right). Thus, the homeodomain of ADNP is required for neuronal differentiation.

### ADNP homeodomain is required for chromatin localization and R-loop suppressor function

ADNP contains both zinc fingers and a homeodomain that can specify DNA binding in vivo. Overexpression studies showed that ADNP lacking the homeodomain, when expressed in ADNP KO mESCs, localizes to some ADNP target genes^57^. In humans, ADNP syndrome is caused by heterozygous mutations in *ADNP*, with recurring mutations (Y719* and R730*) resulting in a truncated protein product lacking the homeodomain^28,60^. To examine whether homeodomain deletion alters the cellular localization of ADNP, we visualized ADNP-KI and ADNPΔHD by immunofluorescence (Supplementary Fig. 6d). We found that in ADNP-KI mESCs, ADNP localizes to DAPI dense regions that correspond to pericentromeres (Supplementary Fig. 6d). However, in ADNPΔHD mESCs, very few cells show strong pericentromeric enrichment (7% compared to 58% in ADNP-KI), and instead the majority (65%) show a general nuclear distribution (Supplementary Fig. 6d). To evaluate whether the homeodomain contributes to chromatin association, we fractionated parental WT, ADNP-KI and ADNPΔHD mESCs into cytosolic, nuclear extract, and nuclear pellet fractions (Fig. 6e). Similar to ADNP in parental mESCs, ADNP in the knock in and homeodomain deletion lines showed nuclear localization and was absent in the cytosolic fraction that contained Tubulin (Fig. 6e). Under our fractionation conditions, in parental and ADNP-KI mESCs, the nuclear fraction of ADNP was predominantly chromatin bound. In contrast, ADNPΔHD showed increased presence in the nuclear soluble fraction and a corresponding decrease in the chromatin-bound fraction (Fig. 6e). EZH2, a component of the Polycomb repressive complex 2 (PRC2) that is unrelated to ADNP, is equally present in the nuclear soluble and chromatin-bound fractions and does not change in ADNPΔHD.

Since deletion of the homeodomain showed a clear change in ADNP’s ability to localize to chromatin, we examined how ADNP homeodomain deletion alters its localization genome-wide. We performed ADNP CUT&RUN in ADNPΔHD mESCs and compared the distribution to ADNP-KI. Peak calling in ADNPΔHD cells identified only 666 peaks, 119 of which overlapped with 12,913 ADNP-KI peaks (Fig. 6f). Strikingly, we found that across the 12,913 ADNP peaks called in ADNP-KI cells, ADNP signal was significantly diminished in ADNPΔHD (Fig. 6g). As an additional validation of our ADNP antibody, we performed CUT&RUN using an antibody to the HA tag in ADNP-KI and ADNPΔHD mESCs. HA CUT&RUN showed specific signal enrichment at ADNP binding sites identified by CUT&RUN with ADNP antibodies, indicating that both HA and ADNP antibodies recognize ADNP-HA V5 protein at the same genomic sites (Supplementary Fig. 6e). We identified only 180 HA peaks that were conserved across two replicates in ADNPΔHD, 152 of which overlapped with 2439 HA peaks called in ADNP-KI (Supplemental Fig. 6f). At HA peaks, both ADNP and HA signal enrichment were significantly decreased in ADNPΔHD (Supplemental Fig. 6g). The reduced ADNPΔHD localization and increased R-loops are clearly seen at the *Sfxn2* gene, which shows ADNP signal enrichment in ADNP-KI but not ADNPΔHD (Fig. 6h). Thus, the homeodomain is required for efficient localization of ADNP to its targets.

Finally, we performed RNA-Seq in ADNPΔHD mESCs and compared to WT and ADNP KO to determine the consequence of homeodomain deletion to gene expression. We performed a comprehensive analysis to identify differentially expressed genes in ADNP KO and ADNPΔHD compared to WT. Most genes in ADNP KO were similarly changed in ADNPΔHD as evidenced by the high positive correlation in fold change compared to WT (Pearson correlation = 0.614) (Fig. 6i). Compared to WT, ADNP KO and ADNPΔHD contained 4694 and 3221 differentially expressed genes, respectively (Fig. 6j, Supplementary Data 2). 1712 genes are significantly deregulated in both ADNP KO and ADNPΔHD (*p* = 3.29e−102, hypergeometric distribution) (Fig. 6j, Supplementary Data 2) and are highly similar in their direction and magnitude of change compared to WT (Supplementary Fig. 7a). These 1712 shared genes include genes that regulate several metabolic, lysosomal, and autophagy pathways that can have consequence to neuronal differentiation and function (Supplementary Data 2). Previous studies showed that loss of ADNP results in upregulation of endoderm genes^63^. Analysis of RNA-seq data showed that the endodermal genes *Lamb1*, *Lamc1*, and *Col4a1* were upregulated in ADNPΔHD similar to ADNP KO (Supplementary Fig. 7b). Therefore, a reason for defective neurodifferentiation in ADNPΔHD could be the incorrect activation of developmental programs that compromise differentiation into neural lineages.

### Rescue of ADNP levels in heterozygous clones enables neuronal differentiation

Mutations that occur in ADNP syndrome occur most frequently in the N terminus of the protein resulting in loss of protein expression from one allele or in the formation of a truncated ADNP protein lacking the homeodomain^28,60^. Our results suggest that homeodomain deletion compromises ADNP localization and that differentiation defects can occur because of reduced protein function. We tested if increasing ADNP protein levels in heterozygous knockout clones (ADNP+/−) can rescue neurodifferentiation defects. We used a dCas9 CRISPR activation system^64^ to design ADNP specific guide RNAs to target the VP64 transcription activator to the endogenous ADNP promoter. Western blot analysis confirmed that ADNP protein was elevated and comparable to WT mESCs in the heterozygous clone expressing dCas9-VP64-sgADNP (called ADNP+/− CRISPRa) (Supplementary Fig. 7c). We differentiated WT, ADNP+/−, and ADNP+/− CRISPRa rescue cell lines into neural progenitors and examined their morphology on days 5 and 6 of differentiation. WT mESCs differentiate normally, spread out, and show cellular extensions (Fig. 6k, top left). However, at the same time point, ADNP+/− cells clump together and show very few extensions (Fig. 6k, top middle). ADNP+/− CRISPRa rescue mESCs resemble WT mESCs and show neurite formation on day 5 (Fig. 6k, top right). On day 6, a much larger proportion of WT and ADNP+/− CRISPRa rescue cells show neurite formation (Fig. 6k, bottom left and right). In contrast, ADNP+/− cells remain sparse and begin to show increased cell death (Fig. 6k, bottom middle). Thus, neural differentiated defects occur as a consequence of ADNP haploinsufficiency and can be corrected by restoring ADNP protein levels.

### Patient-derived ADNP Y719* mutant hiPSCs show R-loop associated CTCF increase at ADNP targets

**Fig. 7:**
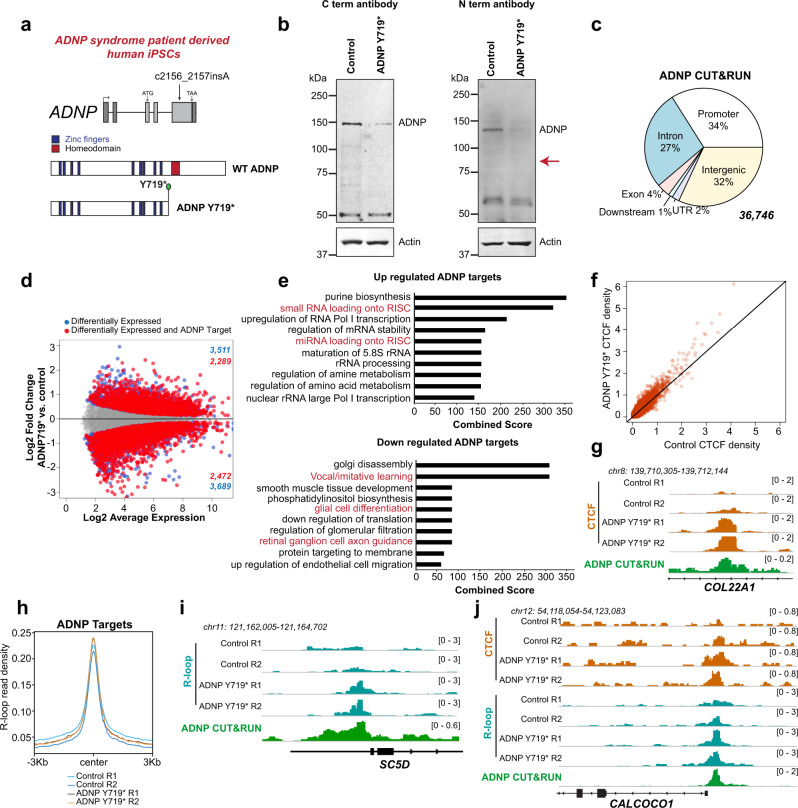
Patient-derived ADNP Y719* hiPSCs display R-loop accumulation and CTCF increase at ADNP targets. **a** Schematic of the human *ADNP* gene showing exons and location of ADNP mutation relative to the cDNA sequence (top). Zinc fingers (blue) and homeodomain (red) are present in WT ADNP protein, while ADNP Y719* lacks the homeodomain (bottom). **b** Western blot for ADNP and Actin in control and ADNP Y719* hiPSCs. ADNP was detected using both C- and N-terminus recognizing ADNP antibodies as indicated. **c** Pie chart displaying distribution of 36,746 hiPSC ADNP peaks across genomic features. **d** MA plot of RNA-Seq expression for 13,057 genes between control and ADNP Y719* hiPSCs. Blue dots indicate differentially expressed genes (adjusted *p*-value <=0.05; *p*-values computed by edgeR with Benjamini–Hochberg adjustment for multiple comparisons). Red dots indicate differentially expressed genes with an ADNP CUT&RUN peak within 3 kb of the gene body. **e** Top 10 most significantly enriched processes (obtained from Enrichr) in differentially expressed ADNP targets (red dots in **d**) that are up- or downregulated in ADNP Y719*. Neurologically relevant processes are shown in red. **f** Scatterplot of CTCF signal (RPM) in control and ADNP Y719* hiPSCs across 36,746 ADNP CUT&RUN peaks called in control hiPSCs. **g** Genome browser view of the *COL22A1* gene showing CTCF CUT&RUN signal (RPM) in control and ADNP Y719* hiPSCs and ADNP CUT&RUN signal (RPM) in control hiPSCs. **h** Signal plot of normalized MapR signal in control and ADNP Y719* hiPSCs over 36,746 ADNP CUT&RUN peaks called in control hiPSCs. **i** Genome browser view of the *SC5D* gene showing MapR signal (RPM) in control and ADNP Y719* hiPSCs and ADNP CUT&RUN signal (RPM) in control. **j** Genome browser view of the *CALCOCO1* gene showing CTCF CUT&RUN and MapR signal (RPM) in control and ADNP Y719* hiPSCs and ADNP CUT&RUN signal (RPM) in control. Source data underlying (**b**) are provided as a Source data file.

Recurring heterozygous nonsense mutation at Y719* is observed in ADNP syndrome patients^28,60^. In ADNP syndrome patients with Y719*, one allele codes for WT protein and the second mutant allele generates a truncated protein product that lacks the homeodomain^65^ (Fig. 7a). Previous reports have suggested that Y719* is a dominant-negative mutation where the truncated protein localizes correctly to its targets^57^. We obtained two hiPSC lines, a mutant line derived from an ADNP syndrome patient with the Y719* mutation and a control from the patient’s mother with wildtype ADNP, both of which were extensively characterized previously^66^. These cells express high levels of the pluripotent markers OCT4 and NANOG and almost undetectable levels of the neural markers NES and PAX6 (Supplementary Fig. 8a). To determine if truncated ADNP is retained in Y719*, we examined ADNP protein levels in control and mutant hiPSCs. We used ADNP antibodies that specifically recognize a region within the N- or C-terminus of ADNP. Western blot analysis showed that ADNP protein levels are decreased in ADNP Y719* compared to control (Fig. 7b). A truncated fragment with a theoretical molecular weight of 80Kda was not detected with the ADNP antibody directed against the N-terminus (Fig. 7b, expected location indicated with a red arrow), suggesting that ADNP syndrome in this case is caused by haploinsufficiency. Examination of RNA-Seq data from control and mutant hiPSCs revealed that ADNP transcript levels were similarly abundant in both lines (Supplementary Fig. 8b), suggesting that ADNP haploinsufficiency in Y719* results from an unstable protein product rather than nonsense-mediated decay of the mRNA. We performed ADNP CUT&RUN in control hiPSCs to evaluate the genomic localization of ADNP (Fig. 7c). ADNP binds to both genic and intergenic regions in hiPSCs (Fig. 7c) with a distribution similar to that observed in mESCs (Fig. 5e). Gene expression analysis by RNA-seq showed that many genes are differentially expressed in ADNP Y719* hiPSCs (Supplementary Data 3), including 4761 ADNP targets that contain an ADNP peak within the promoter or gene body (Fig. 7d). To examine if specific processes were affected in ADNP Y719* hiPSCs, we performed gene ontology analyses of up and downregulated ADNP targets (Supplementary Data 3). Interestingly, deregulated genes in ADNP Y719* hiPSCs were enriched in several neurologically relevant processes such as glial cell differentiation, axon guidance, and vocal learning, and also in microRNA biogenesis pathways that have been implicated in learning and memory (Fig. 7e).

ADNP loss results in increased CTCF binding at many genomic regions^67^. To determine whether a heterozygous Y719* mutation in ADNP can cause CTCF alterations, we performed CTCF CUT&RUN in control and ADNP Y719* hiPSCs. Analysis of CTCF occupancy at ADNP sites showed that CTCF binding was increased at most ADNP sites in ADNP Y719* compared to control (Fig. 7f). One such site is located in the *COL22A1* gene, which shows a clear increased in CTCF across the ADNP binding site in ADNP Y719* (Fig. 7g, Supplementary Fig. 8c).

Our results this far demonstrate that ADNP functions to suppress R-loops at its own binding sites. We asked whether heterozygous ADNP Y719* mutations deregulate R-loops. We performed MapR in control and ADNP Y719* hiPSCs to evaluate whether R-loops are increased at ADNP targets. MapR analysis in ADNP Y719* hiPSCs showed an increase in R-loops at ADNP binding sites (Fig. 7h, i, Supplementary Fig. 8d). Similar to our results from mESCs, R-loops are not changed at regions that do not show ADNP enrichment (Supplementary Fig. 8e). R-loops are consistently increased at all genomic features—promoters, gene bodies, and intergenic sites that exhibit ADNP binding (Supplementary Fig. 8f) and show increase irrespective of transcriptional status of the ADNP target (Supplementary Fig. 8g). Next, we examined whether the increase in R-loops in ADNP Y719* correlated with mistargeting of CTCF. At some regions, including the *CALCOCO1* gene promoter (Fig. 7j), R-loop increases co-occur with gains in CTCF binding. We conclude that heterozygous ADNP mutations that cause ADNP syndrome result in both R-loop and CTCF alterations across the genome.

## Discussion

In this study, we used a proximity labeling-based approach for the in vivo identification of the RNase H proximal proteome (Fig. 1). We identified several known R-loop regulators and demonstrate that ATRX, a chromatin remodeler whose loss results in R-loop accumulation at telomeres^15^, is unable to resolve R-loops in vitro under conditions where both DDX5 and ADNP resolve R-loops (Fig. 2). Instead, we show that the RNA binding activity of ATRX^40,43^ can inhibit R-loop formation, thereby revealing a molecular mechanism for ATRX-mediated R-loop suppression. Importantly, our unbiased proteomic strategy uncovers the presence of homeodomain and zinc finger containing proteins (Fig. 3). Our biochemical characterization of ADNP mechanism at R-loops (Fig. 4), together with the analysis of R-loop dynamics upon ADNP deletion (Fig. 5), revealed a molecular function for ADNP in R-loop suppression. Our results indicate that the zinc fingers of ADNP resolve R-loops, while the homeodomain targets ADNP to chromatin (Fig. 6). ADNP syndrome is caused by a heterozygous mutation in the ADNP gene, most of which would result in protein truncation such that the zinc fingers are retained but not the homeodomain^28,60^. In a previous study, a truncated ADNP protein that contained the 9 zinc fingers was shown to localize efficiently to a few of its target genes, suggesting a dominant-negative mechanism for disease^57^. In contrast, we show that without the homeodomain ADNP cannot localize to chromatin. Importantly, patient-derived hiPSCs that contain ADNP Y719* mutation show severely reduced protein levels and R-loop accumulation at ADNP targets (Fig. 7), arguing for haploinsufficiency as the cause for ADNP syndrome. This mechanistic distinction enables therapeutic strategies based on CRISPR activation technology^68^ that can be used to increase the transcriptional output from the WT ADNP allele.

Although the majority of research efforts to understand mechanisms of R-loop regulation has focused on the helicase family of proteins for their ability to resolve these structures^12,13,22,30,33,69^, several reports also implicate proteins without known helicase activity in R-loop regulation^20,21,70^. Replication protein A (RPA), a single-strand DNA binding protein, is known to localize to R-loop structures in vivo where it stimulates the activity of RNase H to disrupt R-loops^20^. Interestingly, a recent report shows that RPA stabilizes R-loops in vitro through its interactions with RNA^71^, raising the possibility of a context-specific role for this protein in R-loop regulation. The *Arabidopsis* AtNDX homeobox protein binds single-stranded DNA through its homeodomain to stabilize R-loops that in turn inhibit expression of COOLAIR antisense transcripts that regulate flowering^21^. AtNDX contains an atypical and highly divergent homeodomain that is found only in the plant kingdom. Our discovery of a large cohort of homeodomain-containing proteins in our proteome screen (Fig. 3) predicts that this protein domain may have important functional roles at R-loops across species. In addition to homeodomain proteins, we identified a large number of zinc finger proteins (Fig. 3). Zinc finger proteins are a large family of proteins with important roles in development^72^. We speculate that the dual function of zinc finger and homeodomain proteins in transcription activation and repression may be, in part, attributed to their distinct mechanisms at R-loops and to the differential effects R-loops can have in gene regulation. Furthermore, zinc finger proteins bind in a sequence-specific manner to DNA, suggesting that a subset of the large number of zinc finger proteins in the eukaryotic genome may function at discrete locations to regulate R-loop formation. Through their effects on R-loops, zinc finger proteins have the potential to affect localization of epigenetic regulators^73,74^ and architectural proteins^6^ and may therefore play an unappreciated role in the regulation of genome organization.

We uncovered R-loop deregulation at specific sites in an ASD, ADNP syndrome, that correlate with CTCF alterations. Although clearly apparent at some sites, R-loop, and CTCF changes were not strongly correlated across all ADNP sites genome-wide. This could be because Y719* are heterozygous for ADNP mutation or because control and Y719* cell lines, while derived from related individuals, are not isogenic. Alternatively, sequence context or motif strength could determine the extent of R-loop and CTCF co-alteration at some, but not all, ADNP sites. Aberrant R-loops have been implicated in other neurodevelopmental disorders including Fragile X syndrome where R-loops form over expanded CGG repeats^75^. In addition to zinc finger and homeodomain-containing proteins, we also uncovered histone modifiers and transcription factors that are mutated in ASD. These proteins perform diverse nuclear functions and may regulate R-loops at different loci and through distinct mechanisms. Whether R-loop changes that occur in these cases result in defective CTCF localization or if they impact the function of other epigenetic regulators remains to be tested. Our discovery that many potential R-loop interactors are also frequently mutated in ASD indicate that seemingly unrelated neurodevelopmental disorders may share a common thread of deviant R-loops. Thus, development of strategies to resolve anomalous R-loops and correct resultant epigenetic aberrations hold promise for the treatment of a range of neurodevelopmental disorders and cancers.

## Methods

### Cell lines and cell culture

HEK293 cells were cultured in DMEM supplemented with 10% calf serum (Gemini Bio 100510), 1X MEM non-essential amino acids (Gibco 11140), 1X GlutaMAX (Gibco 35050), 25 mM HEPES, 100 U/ml Pen-Strep, and 55 μM 2-mercaptoethanol. E14 mouse embryonic stem cells were cultured on 0.1% gelatin-coated plates in media containing DMEM, 15% fetal bovine serum (Gibco), 1 x MEM non-essential amino acids, 1X GlutaMAX (Gibco 35050), 25 mM HEPES, 100 U/ml Pen-Strep, and 55 μM 2-mercaptoethanol, 3 μM glycogen synthase kinase (GSK) inhibitor (Millipore 361559), 1 μM MEK1/2 inhibitor (Millipore 444966), and LIF (Sigma, ESGRO). Human-induced pluripotent stem cell lines were grown on Geltrex (ThermoFisher A1413302) coated plates in Essential 8 media (Gibco A1517001). *Spodoptera frugiperda* (SF9) insect cell (Expression Systems 94-001S) was cultured in serum-free insect cell culture medium (Expression Systems ESF921).

### Plasmid construction

Turbo biotin ligase was amplified from 3xHA-TurboID-NLS_pCDNA3, a gift from Alice Ting (Addgene plasmid: 107171)^25^ and inserted into BamHI and XhoI sites in pCDNA3. RNaseHΔ was amplified from pICE-RNaseHI-D10R-E48R-NLS-mCherry, a gift from Patrick Calsou (Addgene plasmid: 60367)^76^, digested with KpnI-BamHI enzymes and sub-cloned into pCDNA3-TurboID-NLS-Flag. DDX5 fragment was amplified from HEK293 cDNA and inserted into the BamHI and SalI sites of pGEX-6P-1. ADNP WT and Zn fingers were amplified from ADNP-Strep_flashBAC plasmid^57^ and inserted into SacI and SpeI sites in pFastbac which is modified by inserting Flag tag at the N terminal and His at the C terminal. ADNPΔHD was generated from full-length ADNP-Strep_flashBAC using NEBuilder (NEB E2621S). ADNP homeodomain was amplified from ADNP-strep_flashBAC and inserted into BamHI and SalI sites of pGEX-6P-1. For pET21a-ADNP, ADNP was amplified from ADNP-Strep_flashBAC, digested with SacI and SalI, and sub-cloned into pET21a.

### Generation of ADNP knockout, knock in, and homeodomain deletion cell lines

To generate ADNP knockout, knock in, and homeodomain deletion cell lines with CRISPR/cas9, design of guide RNAs was carried out using the CRISPR Design Tool (https://zlab.bio/guide-design-resources) and inserted into PX459, a gift from Feng Zhang (Addgene plasmid: 62988)^77^ or into lentiCRISPRv2 Blast, a gift from Brett Stringer (Addgene plasmid: 98293)^78^. To generate pCDNA3-ADNP donor plasmid, gBlock gene fragments were synthesized (IDT) and inserted into pCDNA3 using NEBuilder. To generate ADNP knockout cell lines for CRISPRa system, guide RNAs were inserted into CRISPRa-sgRNAs were designed according to Konermann et al.^64^ and inserted into pLentiV2-dCas9-VP64, a gift from Igor Ulitsky (Addgene plasmid: 141104). 1 µg guide RNAs and 1.5 µg donor plasmid were transfected into mESC cell line. ADNP knock in and homeodomain deletions candidate clones were confirmed by PCR using the extracted DNA that was isolated using QuickExtract (Epicentre QE09050), and further confirmed using western blot. All primer sequences can be found in Supplementary Table 1. All cell lines generated in this study are available upon request.

### Proximity labeling by TurboID

TurboID-based proximity labeling assay was performed as described^25^. Cells were incubated with 500 μM biotin (Sigma B4501) for different time points (10, 30, 60 min). Cells were harvested and washed with ice-cold PBS three times to remove extra biotin and incubated on ice for 10 min in 5 volumes buffer A (10 mM Hepes pH 7.9, 5 mM MgCl_2_, 0.25 M sucrose) with 0.1% NP-40. Cells were spun down at 6000 × *g* for 10 min and resuspended in 4 volumes buffer B (10 mM Hepes pH 7.9, 1.5 mM MgCl_2_, 0.1 mM EDTA and 25% glycerol) with 0.42 M NaCl. Nuclear extract was obtained after cells were incubated on ice for 20 min and centrifuged at 9400 × *g* for 15 min. Streptavidin magnetic beads (Thermo 88816) were washed using TBS (25 mM Tris-HCl, pH 7.2, 0.15 M NaCl) containing 0.1% Tween-20 two times and incubated with 1 mg nuclear extract at 4 °C overnight. Streptavidin beads were washed 2 times each with 1% SDS two times and BC500 (50 mM Tris-HCl pH 7.6, 2 mM EDTA, 500 mM KCl), and once with BC100, BC100 containing 2 M urea, and BC100. Each wash step was performed for 5 min at room temperature. Biotinylated proteins were eluted by adding 60 μL 1 x SDS loading buffer and heating at 95 °C 10 min.

### LC-MS/MS analyses and data processing

Liquid chromatography-tandem mass spectrometry (LC-MS/MS) analysis was performed as previously described^79^ using a Q Exactive HF mass spectrometer (ThermoFisher Scientific) coupled with a Nano-ACQUITY UPLC system (Waters). Samples were reduced with TCEP, alkylated with iodoacetamide, digested in-gel with trypsin, and injected onto a UPLC Symmetry trap column (180 μm i.d. × 2 cm packed with 5 μm C18 resin; Waters). Tryptic peptides were separated by reversed-phase HPLC on a BEH C18 nanocapillary analytical column (75 μm i.d. × 25 cm, 1.7 μm particle size; Waters) using a 95 min gradient formed by solvent A (0.1% formic acid in water) and solvent B (0.1% formic acid in acetonitrile). A 30-min blank gradient was run between sample injections to minimize carryover. Eluted peptides were analyzed by the mass spectrometer set to repetitively scan *m*/*z* from 400 to 2000 in positive ion mode. The full MS scan was collected at 60,000 resolution followed by data-dependent MS/MS scans at 15,000 resolution on the 20 most abundant ions exceeding a minimum threshold of 10,000. Peptide match was set as preferred, exclude isotopes option and charge-state screening were enabled to reject singly and unassigned charged ions.

Proteins and peptides were identified using MaxQuant version 1.6.4.0^80^. MS/MS spectra were searched against a UniProt human protein database (10/01/2018) and an in-house contaminants database of common laboratory contaminants, including keratins, bovine proteins detected in FCS, trypsin, and mycoplasma proteins to detect potential mycoplasma contamination of cell cultures. Precursor mass tolerance was set to 4.5 ppm in the main search, and fragment mass tolerance was set to 20 ppm. Digestion enzyme specificity was set to full tryptic specificity with up to two missed cleavages. A minimum peptide length of 7 residues was required for identification. Up to 5 modifications per peptide were allowed; acetylation (protein N-terminal) and oxidation (Met) were set as variable modifications, and carbamidomethyl (Cys) was set as a fixed modification. “Match between runs” feature was not used to transfer identifications across samples. Unique and razor peptides were used for protein quantification. Consensus identification lists were generated with false discovery rates of 1% at protein and peptide levels. Data processing was performed using Perseus version 1.6.8.0 (PMID: 27348712) and Microsoft Excel 2016. Contaminants, reverse, and “only identified by site” identifications were removed from the protein list. In addition, protein entries without any Intensity value in the triplicate experimental and control TurboID groups were removed. Protein intensities were log_2_ transformed and missing values were imputed with a minimal value (log_2_ of 2.16E + 05). Student’s *t*-test *p*-value, *q*-value (*t*-test *p*-value adjusted to account for multiple testing using Benjamini–Hochberg FDR), and log_2_ ratio were calculated using Perseus. Significant protein identifications were defined as proteins detected by a minimum fold change (experimental/control) of 2 and *q*-value less than or equal to 0.05. Additional HeLa, HEK293, and S9.6 IP datasets were downloaded from PRIDE (https://www.ebi.ac.uk/pride/; projects PXD002395 and PXD002960) and re-analyzed using identical MaxQuant parameters described above with the exception that “Match between runs” feature was used for whole proteome samples to minimize missing values due to the complexity of the samples. Published list of significant proteins from Hybrid IP was obtained from ref. ^23^.

### Gene ontology and annotation

To manually assign gene ontology annotations to significant proteins identified by proteomic analyses, lists of human gene symbols belonging to the following GO terms were downloaded from EMBL-EBI QuickGO (https://www.ebi.ac.uk/QuickGO/): “Nucleic acid binding” (GO:0003676; 8497 genes), “cytoskeleton” (GO:0005856; 3298 genes), “transcription regulator activity” (GO:0140110; 2653 genes), “transporter activity” (GO:0005215; 2457 genes), “translation” (GO:0006412; 1645 genes), “chromatin binding” (GO:0003682; 631 genes), and “DNA modification” (GO:0006304; 109 genes). A protein was assigned a GO annotation if its gene symbol was within one or more of these GO terms, and categorized as “Other” if not. For proteins matching more than one GO term, the GO term containing the fewest number of genes was assigned. Enrichment analysis of gene sets was performed with Enrichr^44,45^, a browser-based application that takes a single list of gene symbols as input. The gene set library examined for each enrichment analysis is listed in the heading of Enrichr output tables in Supplementary Data.

### Protein expression and purification

DDX5 was expressed as described previously^81^. Briefly, GST-DDX5-Flag was expressed in Rosetta *E. coli* cell (Millipore 709543) at 16 °C overnight, and purified using GST-agarose beads (Affymetrix 78820) and Anti-FLAG M2 affinity gel (Sigma A2220) as per manufacturer’s instructions. ATRX, ATRXΔRBR, ADNP, ANDPΔHD, and ADNP ZnF were expressed in Sf9 cells. Sf9 cells were infected with baculovirus and harvested after 48hs for protein purification. Cell pellets were lysed in BC300 (50 mM Tris-HCl pH 7.6, 2 mM EDTA, 300 mM KCl, 1 mM β-mercaptotethanol) with 0.1% NP-40 and lysed by sonication. Lysates were spun down and incubated with AntiI-FLAG M2 Affinity Gel. Beads were washed with BC500 (50 mM Tris-HCl pH 7.6, 2 mM EDTA, 500 mM KCl, 1 mM β-mercaptotethanol) and eluted with Flag peptide (Sigma F3290). Proteins were dialyzed, aliquoted, and stored at −80 °C. His tagged full-length ADNP was expressed in BL21 (DE3) (ThermoFisher C601003). Transformed *E. coli* were grown at 37 °C until they reached 0.7–0.8 OD at 600 nm. 0.3 nM IPTG and 200 μM ZnSO_4_ was added to culture and protein expression induced at 18 °C overnight. ADNP-His was purified using Ni-NTA Agarose (QIAGEN 30210) as per manufacturer’s instructions. GST-ADNP homeodomain alone was purified from BL21 *E. coli* after induction at 37 °C for 3 h. Purified proteins were dialyzed against BC100 buffer (50 mM Tris-HCl pH 7.6, 2 mM EDTA, 100 mM KCl, 1 mM β-mercaptotethanol) supplemented with 10 μM ZnSO_4_, aliquoted, and stored at −80 °C.

### ADNP antibody generation and purification

A fragment of human ADNP cDNA corresponding to nucleotides 2860–3309 was cloned into the BamH1 and XhoI sites of pGEX-6P-1 and GST-ADNP fusion protein expressed in BL21 star *E. coli*. GST-ADNP was purified with Glutathione Sepharose® 4B (Fisher Scientific, 45-000-139), followed by on-column cleavage with PreScission Protease. Eluted ADNP antigen was dialyzed into PBS and used for antibody production (Cocalico Biologicals, Inc). ADNP specific antibodies were purified from serum using an ADNP affinity column that was generated by coupling GST-ADNP antigen to Glutathione Sepharose® 4B. The ADNP antibody generated in this study is available upon request.

### Resolution assays

Duplex DNA was formed by mixing equimolar amount of each DNA strand in buffer containing 10 mM Tris pH 7.6, 100 mM NaCl, 1 mM EDTA, heating at 95 °C for 5 min, and slow cooling to 21 °C (Bio-Rad T100 Thermal Cycler). R- or D-loops were assembled by mixing duplex and RNA or ssDNA (1:3 ratio) in buffer containing 90 mM Tris pH 7.5, 90 mM Borate, 10 mM MgCl_2_ (for R-loops), and 40 mM Tris pH 5.5, 10 mM MgCl_2_ (for D-loop) in a total volume of 20 μl for 2 h at room temperature. Excess RNA or ssDNA was removed by purifying assembled R- and D-loops using NucAway spin columns (Ambion AM10070) reconstituted with the same buffer used for assembly.

ATRX, DDX5 R- and D-loop resolution assays were performed as described^14^. Briefly, recombinant proteins (amounts as indicated in figure legends) were incubated with 1 nM R- and D-loop substrates in buffer containing 25 mM morpholinepropanesulfonic acid, pH 7.0, 60 mM KCl, 5 mM MgCl_2_, 0.2% Tween-20) with 2 mM DTT and 5 mM ATP in a total volume of 20 μl at 37 °C for 20 min. Reactions were stopped by addition of 4 μl stop buffer (20 mM Tris-HCl, pH 7.5 and 2 mg/ml proteinase K) and incubated at 37 °C for 20 min. Reactions were resolved at 4 °C on an 8% native acrylamide gel in 0.5X TBE running buffer supplemented with 1 mM MgCl_2_. ADNP resolution assays were performed in binding buffer (50 mM Tris pH 8.0, 100 mM NaCl, 10 μg/ml BSA, 1 mM DTT, 0.1 mM EDTA, and 5% Glycerol) containing 2 μg yeast tRNA (Ambion AM7119) at 30 °C for 20 min.

To test effects of specific proteins on R- or D-loop formation, purified proteins (as specified in figures) were incubated with 60 nM RNA or ssDNA in 10 μL total volume in buffer containing 50 mM Tris pH 8.0, 100 mM NaCl, 1 mM DTT, 0.25 mg/ml BSA, 0.5% Glycerol, and 1.5 mM MgCl_2_ at room temperature for 20 min. Protein-nucleic acid complexes were added to 20 nM duplex DNA and incubated at room temperature in 90 mM Tris pH 7.5, 90 mM Borate, 10 mM MgCl_2_ (for R-loops) and 40 mM Tris pH 5.5, 10 mM MgCl_2_ (for D-loop) for 2 h. Reactions were stopped by addition of 4 μl stop buffer (20 mM Tris-HCl, pH 7.5 and 2 mg/ml proteinase K) and incubated at 37 °C for 20 min. Reactions were resolved on an 8% native acrylamide gel in 0.5X TBE running buffer supplemented with 1 mM MgCl_2_ at 120 V for 2 h at 4 °C. Results were visualized using Amersham Typhoon Gel and Blot Imaging Systems (GE) and quantified using ImageJ. Sequences of all oligonucleotides used can be found in Supplementary Table 1.

### S9.6 dot blot

Genomic DNA extraction was performed as described in ref. ^82^. 5 × 10^6^ cells were harvest, washed in 1XPBS, and resuspended in 1.6 ml Tris-EDTA (TE) buffer containing 41.5 μl of 20% SDS and 5 μl of proteinase K (20 mg/ml) and incubated at 55 °C overnight. DNA was extracted with phenol-chloroform (Sigma P3803) and precipitated with ethanol. Genomic DNA was digested with 50 U of HindIII, EcoRI, BsrGI, and SspI overnight. Different concentrations of DNA were loaded on Amersham Hybond-N+ (GE Healthcare RPN203B) using a Minifold I system apparatus (Cytiva 10447900). Membrane was washed with 2x SSC (300 mM NaCl, 30 mM sodium citrate) and crosslinked in a UV Stratalinker (0.125 J/cm^2^). The membrane was blocked with 5% non-fat milk, incubated with S9.6 antibody (1.5 μg/ml) overnight at 4 °C, and processed similar to western blots, and visualized on an Odyssey Infrared Imager (LI-COR). After visualization, membrane was washed with stripping buffer (100 mM Tris pH 6.8, 2 M NaCl, 100 mM β-mercaptoethanol, 2% sarkosyl) and probed with dsDNA antibody (1:1000, Abcam ab27156) to confirm equal loading. Antibodies used in this study can be found in Supplementary Table 2.

### DRIP-qPCR

DRIP was performed as previously described^5,82^. 5 million mESCs were harvested, washed with PBS, and resuspended in 1.6 ml TE buffer with 41.5 μL of 20% SDS and 5 μL of Proteinase K (20 mg/ml) and incubated at 55 °C overnight. Genomic DNA was isolated using phenol/chloroform and ethanol precipitation. DNA fragmentation was performed at 37 °C overnight using 50 U restriction enzymes (HindIII, EcoRI, BsrGI, XbaI, SspI) with 2 mM spermidine. Half of the DNA was treated with 3 μL RNase H (NEB M0297L) overnight as a negative control. Digested DNA (5 μg) was incubated overnight with 10 μg S9.6 antibody (Supplementary Table 2) in 500 μL binding buffer (10 mM Na_2_HPO_4_, 140 mM NaCl, 0.05% Triton X-100) at 4 °C. DNA/antibody complexes were enriched using 20 μL Dynabeads Protein G (Invitrogen 10004D). After three washes, the immunoprecipitated DNA was eluted with 100 μL elution buffer (50 mM Tris pH 8.0, 10 mM EDTA, 0.5% SDS) containing 7 μL Proteinase K (20 mg/ml) at 55 °C for 45 min. DNA was purified using phenol/chloroform and ethanol precipitation and quantified with qPCR (BIO-RAD CFX Connect Real-time System). The primer sequences are provided in Supplementary Table 1.

### Cell fractionation and immunofluorescence

Nuclear fractionation was performed as described previously in ref. ^43^. Briefly, 10 million cells were washed with 1 ml PBS and resuspended in 600 μL Buffer A (10 mM HEPES pH 7.9, 5 mM MgCl_2_, and 0.25 M sucrose) containing 0.1% NP-40 and incubated on ice for 10 min. Cells were centrifuged at 6000 × *g* at 4 °C for 10 min. The supernatant (cytosol) was transferred to a new tube, and cell pellet was resuspended in 250 μL Buffer B (10 mM HEPES pH 7.9, 0.1 mM EDTA, 1.5 mM MgCl_2_, and 25% glycerol). 20 μL of 2.5 M KCl was added to 80 μL of cell pellet resuspension for a final concentration of 500 mM KCl and treated as total nuclear extract. The remaining 170 μL was brought to 300 mM KCl by addition of 2.5 M KCl and incubated on ice for 15 min. The nuclei were centrifuged at 9400 × *g* at 4 °C for 15 min and supernatant was transferred to new tubes as the nuclear-soluble fraction. The nuclear pellet was resuspended in 70 μL Buffer B containing 1 M KCl and lysed on ice for 20 min. 130 μL Buffer B was added to reduce salt concentration, and sonicated. The pellet fraction was centrifuged at 9400 × *g* for at 4 °C for 15 min. Ten percent of each fraction was used for western blot. Immunofluorescence was performed as previously in ref. ^83^. Antibodies used in this study can be found in Supplementary Table 2.

### Mouse embryonic stem cell differentiation into neural progenitor cells

Differentiation of mESCs to NPCs was performed as previously described^61^. mESCs were plated into gelatin-coated wells of a 6-well plate (30,000 cells per well) in mESC medium (see Cell culture) and cultured overnight to allow attachment to the plate. To induce differentiation, mESC medium was withdrawn and N2B27 medium (50% Neurobasal medium, 50% DMEM/F-12 medium, 1 mM sodium pyruvate, 0.1 mM non-essential amino acids, 2 mM L-Glutamine, 0.5% Pen-Strep, 55 µM beta-mercaptoethanol, 40 µG/mL bovine serum albumin, 1x N-2 supplement, 1x B-227 supplement) containing 10 ng/mL human basic fibroblast growth factor (bFGF, Gemini Bio #300-112 P) was added. Media was replaced with N2B27 medium containing bFGF at 24 and 48 h after induction. At 72 and 96 h after induction, media was replaced with N2B27 medium containing 500 nM smoothened agonist (SAG, Sigma #566661). Cells were imaged at 120 h after induction (day 5).

### MapR, CUT&RUN, and RNA-Seq

MapR was performed as previously described^51,52^ with heterologous *Drosophila* DNA added as a spike-in control. Briefly, 10 million cells were harvested and washed with 1.5 ml wash buffer (20 mM HEPES pH 7.5, 0.15 M NaCl, 0.5 mM spermidine, and 1 mM protease inhibitors) two times. Cells were immobilized on Concanavalin A-coated beads by rotating at room temperature for 1 h, then divided equally into two tubes. Cells were resuspended in 50 μL Dig-wash buffer (20 mM HEPES pH 7.5, 0.15 M NaCl, 0.5 mM spermidine, 1 mM protease inhibitors, and 0.02% Digitonin) and GST-MNase or GST-RHΔ-MNase was added to a final concentration of 1 μM. Beads were rotated at 4 °C overnight, then washed 3 times using Dig-wash buffer and resuspended in 100 μL Dig-wash buffer. For MNase activation, CaCl_2_ was added to a final concentration of 2 mM and beads were incubated in wet ice at 0 °C for 30 min. 2x STOP buffer (340 mM NaCl, 20 mM EDTA, 4 mM EGTA, 0.02% Digitonin, 5 μg RNase A, 5 μg linear acrylamide and 1 ng/ml spike-in DNA) was added to stop reaction. Cells were incubated at 37 °C for 10 min to facilitate the release of digested DNA fragments, centrifuged at 4 °C at 16,000 × *g* for 5 min, and supernatants transferred to new tubes. 2 μL 10% SDS and 5 μg proteinase K were added and samples were incubated at 70 °C for 10 min. DNA was purified using phenol/chloroform and ethanol precipitation.

CUT&RUN was performed as previously described^53,54^ using ADNP antibody, CTCF antibody (Cell Signaling 3418S), and HA antibody (Roche 11583816001) and with heterologous *Drosophila* DNA added as a spike-in control. Briefly, 5 million cells were washed three times and resuspended with 1 ml wash buffer. 10 μL of activated Concanavalin A-coated beads were added and samples incubated at room temperature for 1 h by rotating. Cells were resuspended in 50 μL Dig-wash buffer containing 2 mM EDTA. ADNP antibody (10 μg), CTCF antibody (5 μg), or HA antibody (5 μg) was added in the indicated amounts and samples were rotated overnight at 4 °C. 5 μg Rabbit IgG (Sigma I5006) was added to a non-specific control sample. For HA antibody, beads were washed once with Dig-wash buffer and incubated with rabbit anti-mouse IgG (ThermoFisher SA5-10192) at 4 °C for 1 h as a secondary antibody. Cells were washed once with Dig-wash buffer and resuspended in 50 μL Dig-wash buffer. Protein A-MNase was added to a final concentration of 700 ng/ml and samples were incubated at 4 °C for 1 h. Cells were washed with Dig-wash buffer twice and resuspended in 100 μL Dig-wash buffer. For MNase activation, CaCl_2_ was added to a final concentration of 2 mM and beads were incubated in wet ice at 0 °C for 30 min. 100 μL 2x STOP buffer was added to stop reaction. Cells were incubated at 37 °C for 10 min to facilitate the release of digested DNA fragments, centrifuged at 4 °C at 16,000 × *g* for 5 min, and supernatants transferred to new tubes. 2 μL 10% SDS and 5 μg proteinase K were added and samples were incubated at 70 °C for 10 min. Antibodies used in this study can be found in Supplementary Table 2.

RNA samples were extracted using Trizol reagent (Invitrogen) and subjected to DNase digestion with Turbo DNase (Ambion AM2238). RNA samples were then rRNA-depleted using FastSelect -rRNA HMR (Qiagen) and converted to cDNA using Ultra II Directional RNA Library Prep Kit (NEB E7760).

DNA and cDNA samples were end-repaired using End-Repair Mix (Enzymatics), A-tailed using Klenow exonuclease minus (Enzymatics), purified with MinElute columns (Qiagen), and ligated to Illumina adapters (NEB #E7600) with T4 DNA ligase (Enzymatics). Size selection for fragments >150 bp was performed with AMpure XP (Beckman Coulter). Libraries were PCR amplified with barcoded adapters for Illumina sequencing (NEB #E7600) using Q5 DNA polymerase (NEB #M0491) and purified with MinElute. Sequencing was performed on a NextSeq 500 instrument (Illumina) with 38 × 2 paired-end cycles.

### Sequencing analysis

MapR data was processed as described earlier^84^. CUT&RUN and MapR reads were mapped to the mouse genome (mm10) or human genome (hg19) with Bowtie2 version 2.2.9^85^ using default parameters and paired-end setting. Peaks were called for each sample using MACS2 2.2.1^86^ using the parameters “--broad --broad-cutoff 0.1 -f BAMPE -g mm/hs --keep-dup all” for MapR and “-f BAMPE -g mm/hs --keep-dup all” for CUT&RUN. Signal plots were generated using the computeMatrix and plotProfile functions in deepTools version 3.4.1^87^. Read density values used for scatterplots and boxplots were calculated using the multiBigwigSummary function in deepTools. RPM BigWig tracks were generated using the bamCoverage function in deepTools using the parameters “--binSize 5 --extendReads --normalizeUsing CPM –blackListFileName”, which removes a known set of ENCODE blacklist regions^88^. Normalized R-loop signal BigWig tracks were generated by subtracting the GST-MNase RPM signal of a sample from the corresponding MapR RPM signal using the bigwigCompare function in deepTools and parameters “--operation subtract --binSize 5”.

Differential R-loop and CUT&RUN analysis were performed in R version 3.6.1 using the DiffBind package, version 2.12.0 (http://bioconductor.org/packages/release/bioc/html/DiffBind.html). Peaks present in at least two samples and that were not in an unknown contig or blacklisted region were kept for analysis, and differential occupancy was called using the edgeR method and an FDR cutoff of 0.05. Overlap between peaksets was defined through simple genomic overlap between regions. Enrichment *p*-values of overlapping peaks were obtained using the hypergeometric test.

RNA-Seq data were aligned using STAR version 2.7.3^89^. RSEM version 1.3.3^90^ was used to obtain estimated counts. Differential analysis of RNA-Seq data was performed in R using the packages limma version 3.40.6 and edgeR version 3.26.8. For RNA-Seq differential analysis, genes were filtered using the edgeR built-in function “filterByExpr”. Annotation of ADNP peaks was performed using ChIPseeker version 1.20.0^91^. An ADNP peak was associated with a target gene if it was annotated as being either inside the gene body, or within 3 kb upstream or downstream of the gene.

### Statistics and reproducibility

Student’s two-sided *t*-tests, Welch’s two-sided *t*-tests, and hypergeometric tests were performed as described in “Results” and figure legends. Adjusted *p*-values obtained from edgeR and Enrichr analyses were computed by the respective softwares including correction for multiple comparisons. All western blots, silver, and Coomassie blue stains, resolution assays, and differentiation experiments were repeated at least three times to confirm similar results and ensure reproducibility.

### Reporting summary

Further information on research design is available in the Nature Research Reporting Summary linked to this article.

## Supplementary information

Supplementary Information

Description of additional Supplementary File

Supplemental data 1

Supplemental data 2

Supplemental data 3

Reporting Summary

## Data Availability

The data that support this study are available from the corresponding author upon reasonable request. All mass spectrometry raw data generated in this study have been deposited to the MassIVE public repository under accession code MSV000087568 and the ProteomeXchange repository under accession code PXD026473. MapR, CUT&RUN, and RNA-Seq sequencing data and processed tracks generated in this study have been deposited in the NCBI GEO database under accession code GSE171401. RNA-Seq data for WT mESCs used in this study is available in the NCBI GEO database under accession code GSE160578. Source data are provided with this paper.

## Source data

Source Data

## Code availability

All code used to generate figures, tables, and/or analysis in this paper is available upon request.
