# Supplementary material for: Proximity labeling identifies a repertoire of site-specific R-loop modulators
Source: Nat Commun. 2022 Jan 10;13:53. doi: 10.1038/s41467-021-27722-6 (PMC8748879; doi:10.1038/s41467-021-27722-6)
Supplement: Supplementary file 1 — Supplementary Information [file 41467_2021_27722_MOESM1_ESM.pdf]

SUPPLEMENTARY INFORMATION FOR:

Proximity labeling identifies a repertoire of site-specific R-loop modulators

Qingqing Yan[1,2ξ], Phillip Wulfridge[1,2ξ], John Doherty[1,2], Jose L. Fernandez-Luna[3,4], Pedro J. Real[5,6], Hsin-Yao Tang[7], Kavitha Sarma[1,2*]

1. Gene Expression and Regulation Program, The Wistar Institute, Philadelphia PA 19104
2. Epigenetics Institute, University of Pennsylvania, Philadelphia PA 19104
3. Genetics Unit, Hospital Valdecilla, 39008 Santander, Spain
4. Instituto de Investigación Valdecilla (IDIVAL), 39012 Santander, Spain
5. Gene Regulation, Stem Cells and Development Group, Department of Genomic Oncology, GENYO: Centre for Genomics and Oncological Research-Pfizer, University of Granada, Junta de Andalucía, PTS, 18016 Granada, Spain
6. Department of Biochemistry and Molecular Biology I, Faculty of Science, University of Granada, 18016 Granada, Spain.
7. The Wistar Institute, Philadelphia PA 19104

[ξ] These authors contributed equally

* Corresponding author: kavitha@sarmalab.com

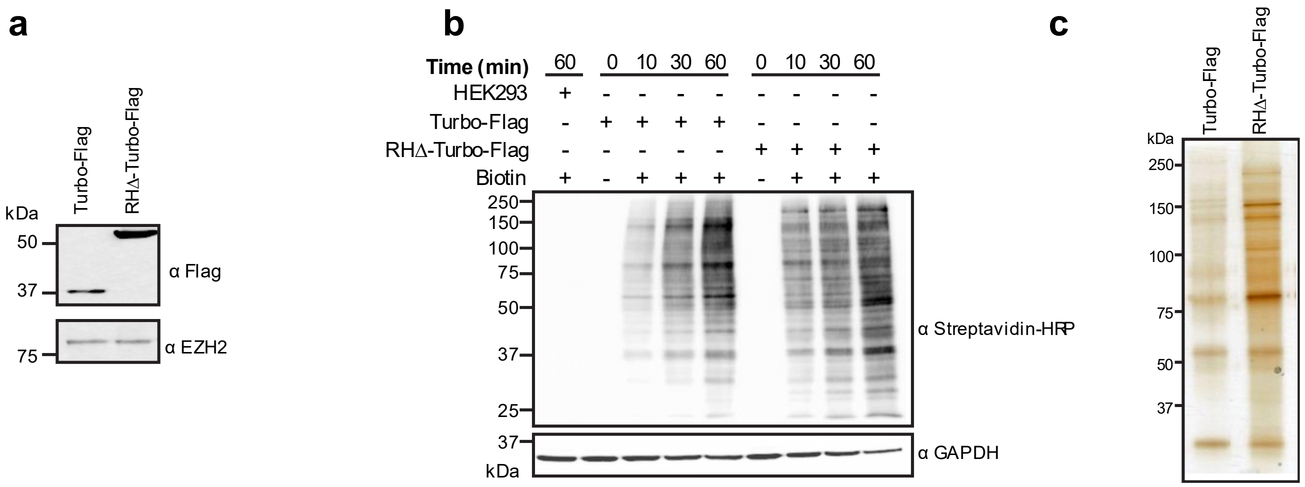

Supplementary Figure 1: In vivo RNase H mediated proximity labeling uncovers R-loop regulators.

(a) Western blot for Turbo-Flag and RHΔ-Turbo-Flag expression in HEK293. Antibodies used are indicated on the right.

(b) Analysis of biotinylation efficiency in vivo in HEK293 TurboID and RHΔ-TurboID. Cells were treated with biotin for the indicated times, nuclear extract prepared, and analyzed by western blot using anti-streptavidin HRP. GAPDH serves as a loading control.

(c) Silver stain of streptavidin pull-down from HEK293 TurboID and RHΔ-TurboID.

Source data underlying Supplementary Fig. 1a-c are provided as a Source Data file.

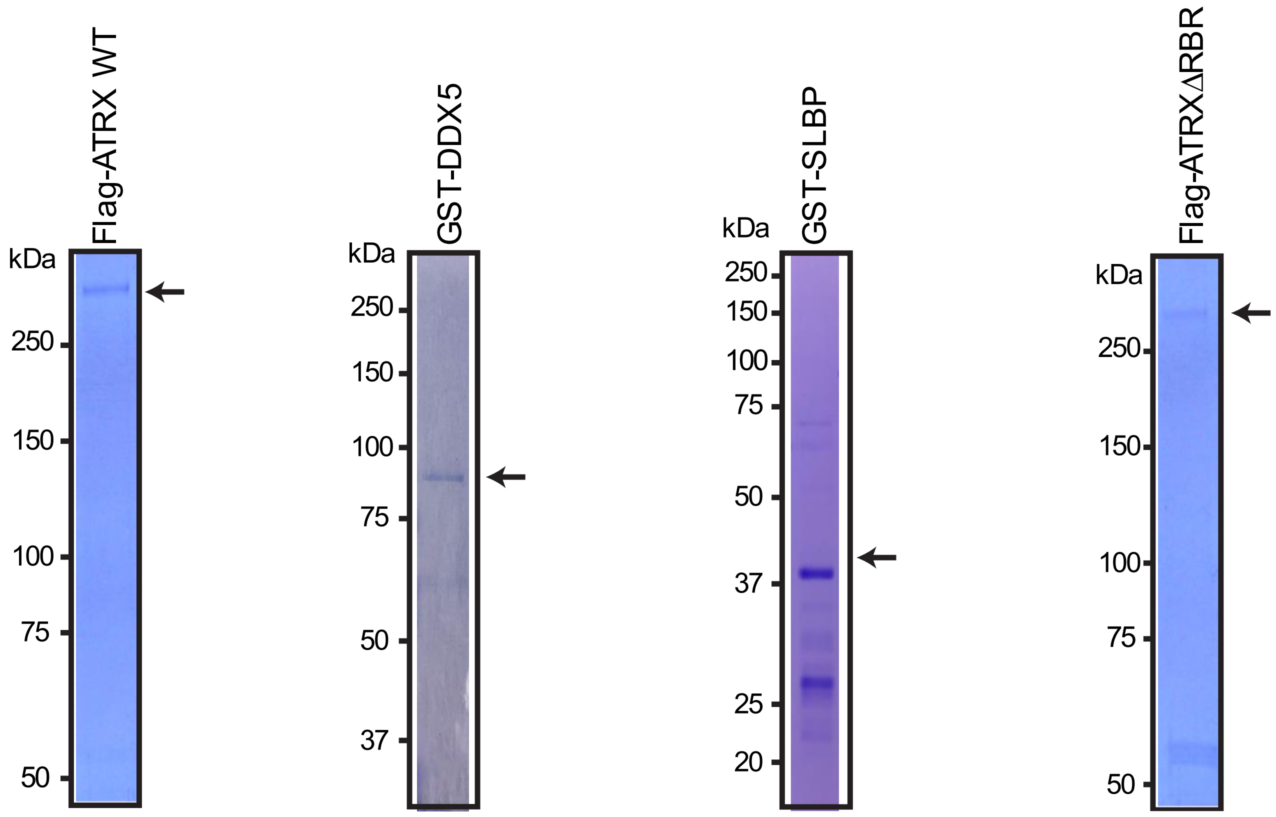

Supplementary Figure 2: Coomassie blue stain for purified ATRX WT, DDX5, SLBP and ATRXΔRBR.

ATRX WT and ATRXΔRBR proteins from Sf9 cells and GST-DDX5 and GST-SLBP from E.coli. Proteins are indicated with black arrowhead. Source data are provided as a Source Data file.

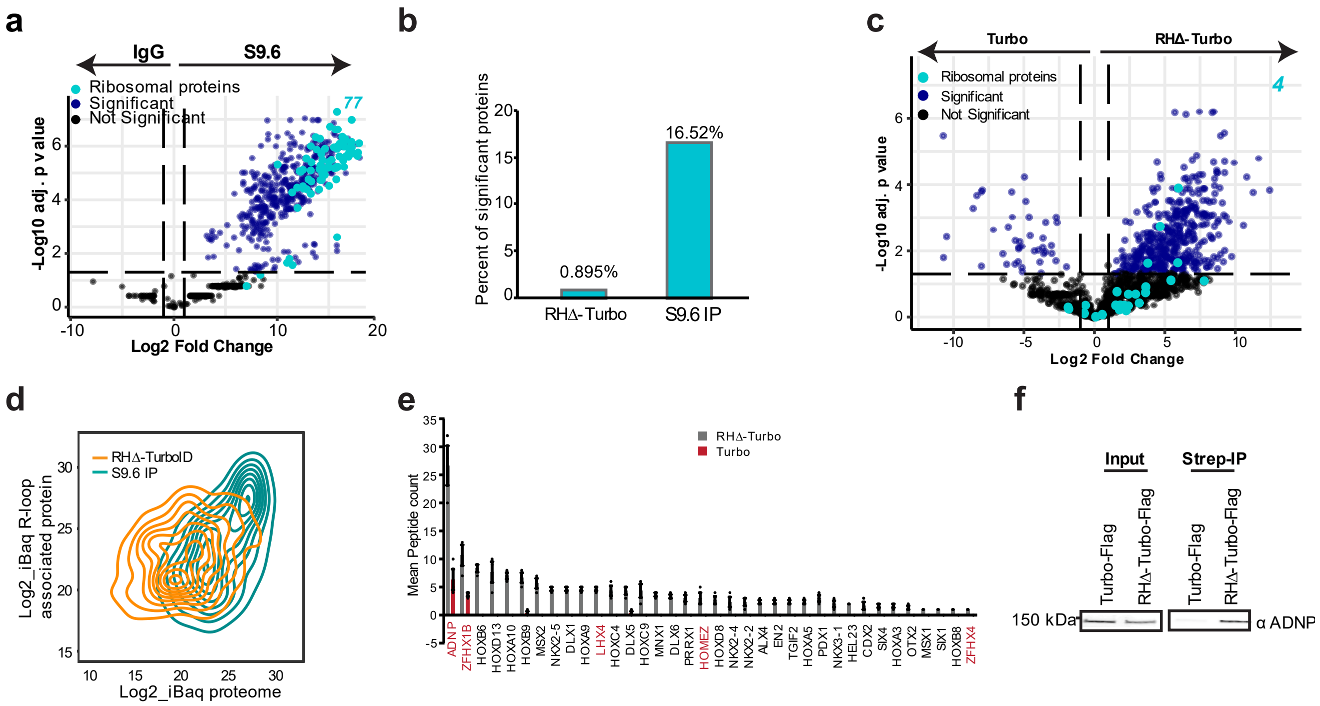

Supplementary Figure 3: In comparison to S9.6 IP, RHΔ-TurboID does not enrich for non-specific ribosomal proteins.

(a) Volcano plot of S9.6 IP showing log2 fold changes in protein intensities on the x-axis and -log10 adjusted p-values (Student's two-sided t-test with Benjamini-Hochberg adjustment for multiple comparisons) on the y-axis. Significantly enriched proteins (blue, $p<0.05$), non-significant (black), enriched ribosomal proteins (cyan).

(b) Ribosomal proteins shown as a percent of significantly enriched proteins from RHΔ-TurboID and S9.6 IP.

(c) Volcano plot of RHΔ-TurboID showing log2 fold changes in protein intensities on the x-axis and -log10 adjusted p-values (Student's two-sided t-test with Benjamini-Hochberg adjustment for multiple comparisons) on the y-axis. Significantly enriched proteins (blue, adjusted p-value <0.05), non-significant (black), enriched ribosomal proteins (cyan).

(d) Contour density plot showing enrichment of R-loop interactors from RHΔ-TurboID (orange) and S9.6 IP (teal) relative to their abundance in the whole proteome. RHΔ-TurboID and S9.6 IP were compared to HEK293 and HeLa proteomes respectively.

(e) Mean peptide counts of all significantly enriched (adjusted p-value < 0.05, Student's two-sided t-test with Benjamini-Hochberg adjustment for multiple comparisons) homeodomain proteins from

3 independent RHΔ-TurboID experiments. Proteins containing Zinc finger are labeled with red. TurboID alone (red), RHΔ-TurboID (black). Error bars indicate +/- SEM.

(f) Western blot for ADNP in HEK293 Turbo and RHΔ-Turbo input (left) and in streptavidin pull-downs (right). ADNP are detected with anti-ADNP antibodies. Source data are provided as a Source Data file.

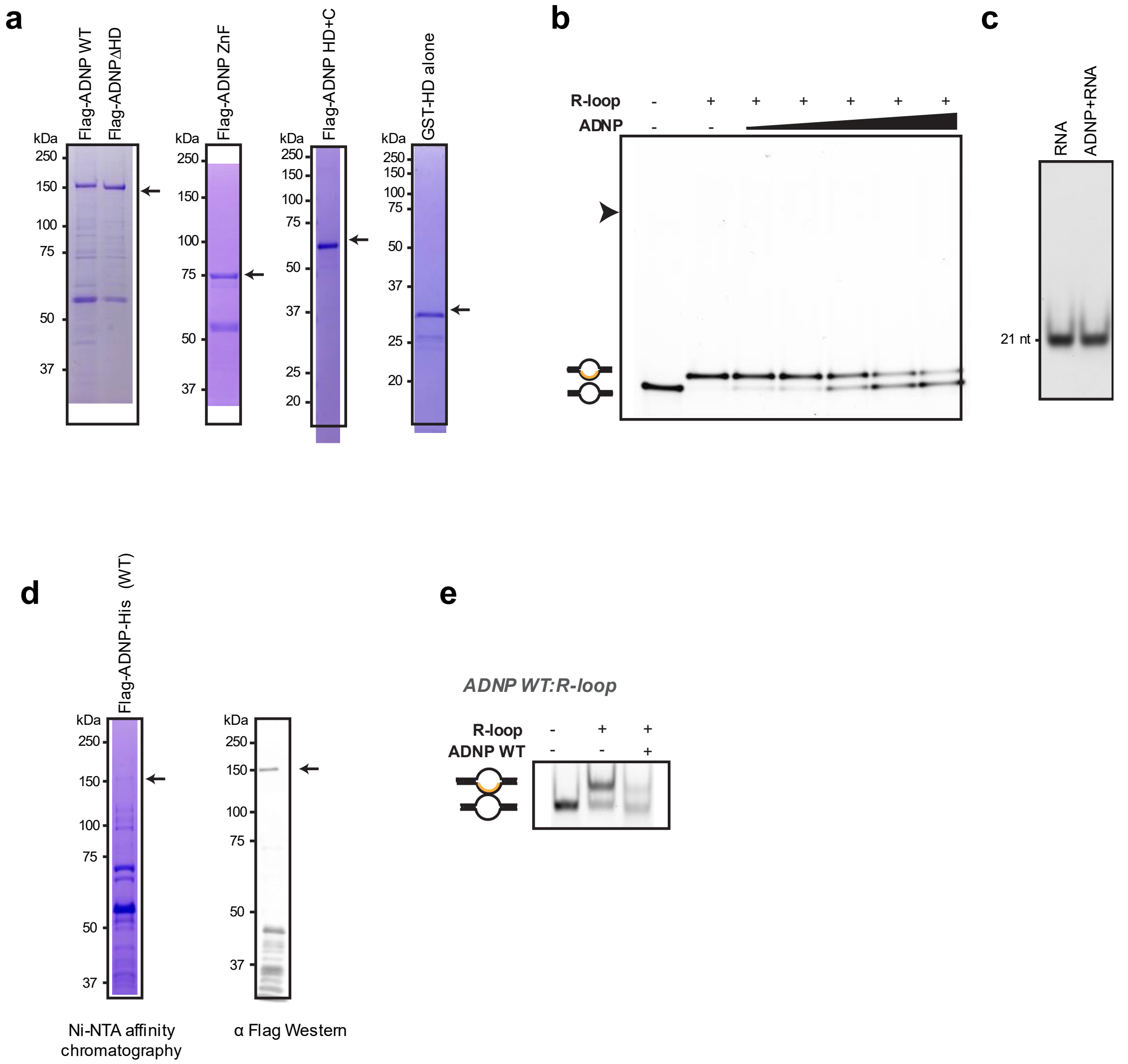

Supplementary Figure 4: Full length ADNP resolves R-loops in vitro

(a) Coomassie blue stain for purified ADNP WT, ADNPΔHD, ADNP Zinc fingers (ADNP ZnF) proteins from Sf9 cells, ADNP homeodomain and C terminus (ADNP HD+C) and GST-ADNP homeodomain alone from E.coli. Proteins were indicated with black arrowhead.

(b) EMSA with ADNP (10, 20, 40, 80, and 160 nM) and 1 nM R-loop substrates. Expected position of ADNP-R-loop complex is indicated (black arrowhead).

(c) RNA degradation assay to test RNase activity in ADNP WT protein. ADNP WT (200 nM) and RNA (1nM) are used.

(d) Coomassie blue stain for purified ADNP WT from E. coli (left). Western blot of purified bacterial ADNP WT using anti-Flag antibody (right). Full length protein is indicated with black arrowhead.

(e) R-loop resolution assay with 25 nM ADNP WT purified from bacteria and 1 nM R-loop substrates. Positions of duplex and R-loops are shown.

Source data underlying Supplementary Fig. 4a-e are provided as a Source Data file.

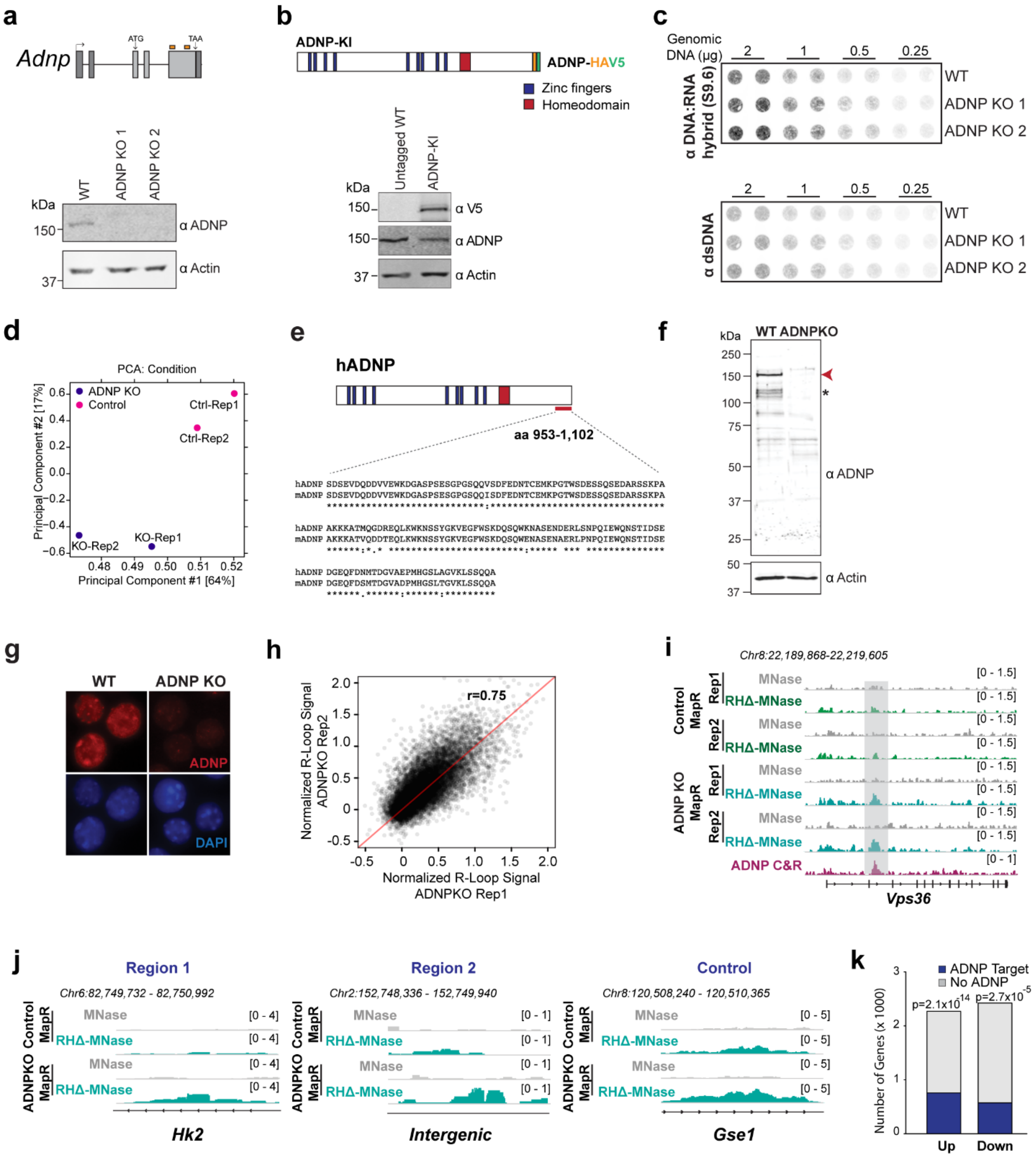

Supplementary Figure 5: ADNP deletion does not result in global R-loop deregulation.

(a) Schematic of *Mus musculus Adnp* gene showing the location of gRNAs (orange) used to generate ADNP KO (top). Western blot of ADNP and actin in WT mESCs and two ADNP knock out clones (bottom). Antibodies used are indicated on the right.

(b) Schematic of HA and V5 epitope tagged ADNP-KI showing zinc fingers in blue and homeodomain in red (top). Western blot for V5 and ADNP in parental WT and ADNP-KI mESCs (bottom). Actin serves as loading control. Antibodies indicated on the right.

(c) Dot blots for DNA:RNA hybrids using S9.6 antibody (top) and dsDNA (bottom) in WT ESCs and ADNP KO clones. DNA amounts (μg) used are indicated on top.

(d) Principal component analysis of normalized R-loop signal in control and ADNP KO mESCs across 61,652 R-loop peaks called across control and ADNP KO samples.

(e) Schematic of hADNP protein showing the C-terminal fragment used to generate ADNP antibody (top). Comparison of human and mouse protein sequences at the ADNP C-terminus (bottom).

(f) Western blot of ADNP and Actin in WT and ADNP KO mESCs. ADNP C-terminus antibody was used. Antibodies used are indicated on the right. Source data are provided as a Source Data file.

(g) Immunostaining of WT and ADNP KO mESCs with ADNP C-terminus antibody (red). Source data are provided as a Source Data file.

(h) Scatter plot of normalized MapR signal between replicates 1 and 2 of ADNP KO mESCs across 12,913 ADNP CUT&RUN peaks called in control mESCs. Pearson correlation=0.75.

(i) Genome browser view of the *Vps36* gene showing MapR signal (RPM) in control and ADNP KO mESCs and ADNP CUT&RUN signal (RPM) in control mESCs. ADNP peak highlighted in grey.

(j) Genome browser tracks of the *Hk2* gene, an intergenic region, and the *Gse1* gene showing MapR signal (RPM) in control and ADNP KO mESCs.

(k) Number of ADNP target (blue) and non-ADNP target genes (grey) differentially expressed (adjusted p-value <=0.05; p-values computed by edgeR with Benjajmini-Hochberg adjustment for multiple comparisons) between WT and ADNP KO mESCs. P-values, over-enrichment (Up) or under-enrichment (Down) of ADNP target genes compared to background, hypergeometric test.

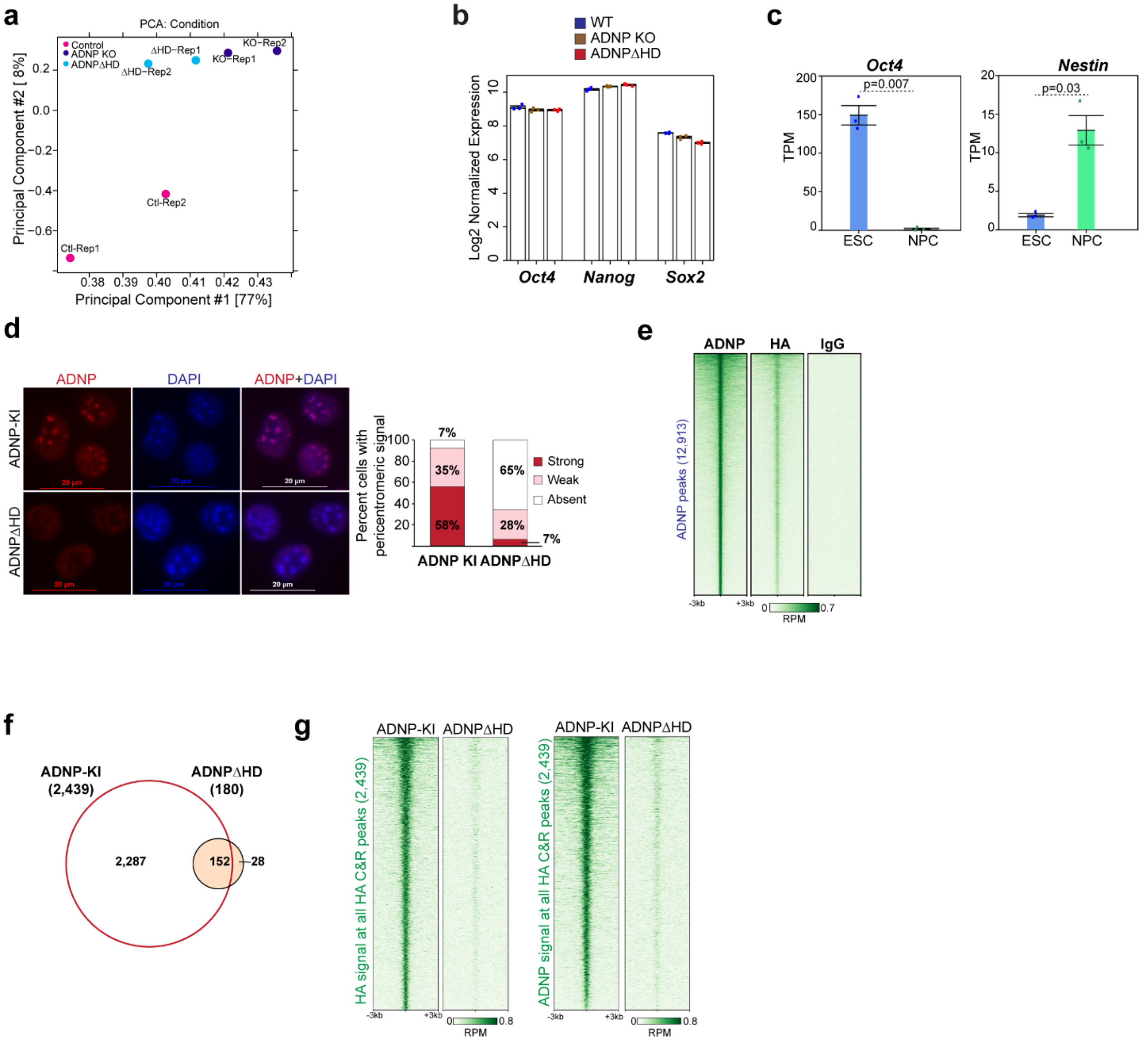

Supplementary Figure 6: ADNP homeodomain deletion protein does not localize to ADNP targets.

(a) Principal component analysis of normalized R-loop signal in control (ADNP-KI), ADNP KO, and ADNPΔHD mESCs across 12,913 ADNP CUT&RUN peaks.

(b) Bar chart of log2 normalized RNA-Seq showing expression of *Oct4*, *Nanog,* and *Sox2* in WT, ADNP KO, and ADNPΔHD mESCs. Bar chart, mean +/- SEM; individual values shown as dots (n = 3 biologically independent samples).

(c) Bar chart of RNA-Seq TPM showing expression of *Oct4* (left) and *Nestin* (right) in WT mESCs and WT NPCs. Bar chart, mean +/- SEM; individual values shown as dots. p, Welch's two-sided t-test (n = 3 biologically independent samples).

(d) Immunostaining of ADNP-KI and ADNPΔHD-HAV5 mESCs with V5 antibody (red). Quantification shows percent cells (n=200) with strong, weak, or absent pericentromeric signal. Scale bar=20 μm. Source data are provided as a Source Data file.

(e) Heatmap of ADNP, HA, and IgG CUT&RUN signal in ADNP-KI mESCs across 6-kb windows centered on ADNP CUT&RUN peaks. Rows are sorted in decreasing order by mean signal across all samples. RPM, reads per million.

(f) Venn diagram showing overlap between 2,439 HA CUT&RUN peaks called in two ADNP-KI replicates and 180 HA CUT&RUN peaks called in two ADNPΔHD replicates.

(g) Heatmap of HA (left) and ADNP (right) CUT&RUN signal (RPM) in ADNP-KI and ADNPΔHD across 6-kb windows centered on HA peaks called in ADNP-KI. Rows are sorted in decreasing order by mean signal across all samples.

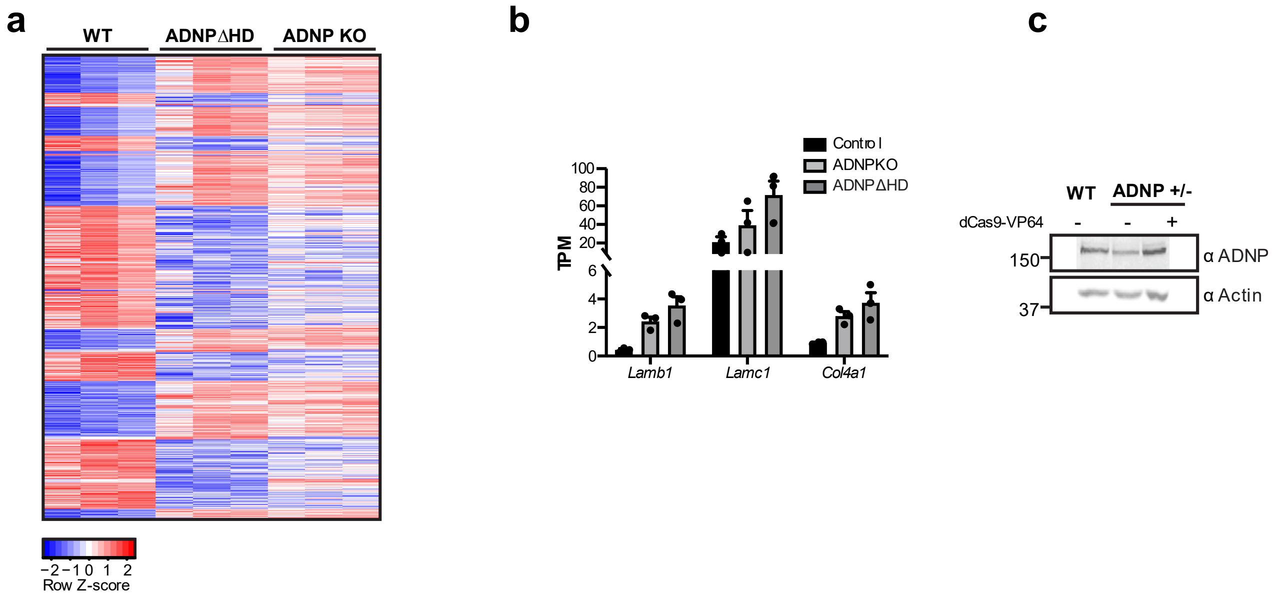

Supplementary Figure 7: ADNP homeodomain deletion and ADNP knock out similarly deregulate shared genes.

(a) Heatmap of RNA-Seq data for 1,712 genes differentially expressed in both ADNP KO and ADNPΔHD mESCs, showing normalized expression (z-scores) for each biological replicate in WT, ADNP KO, and ADNPΔHD. Rows and columns are sorted by unsupervised hierarchal clustering.

(b) Bar chart of RNA-Seq TPM showing expression of *Lamb1*, *Lamc1* and *Col4a1* in WT, ADNP KO, and ADNPΔHD mESCs. Bar chart, mean + SEM; individual values shown as dots (n = 3 biologically independent samples). Source data are provided as a Source Data file.

(c) Western blot for ADNP in WT, ADNP$^{+/-}$ and ADNP$^{+/-}$ CRISPRa mESCs. Antibodies used are indicated on the right. Source data are provided as a Source Data file.

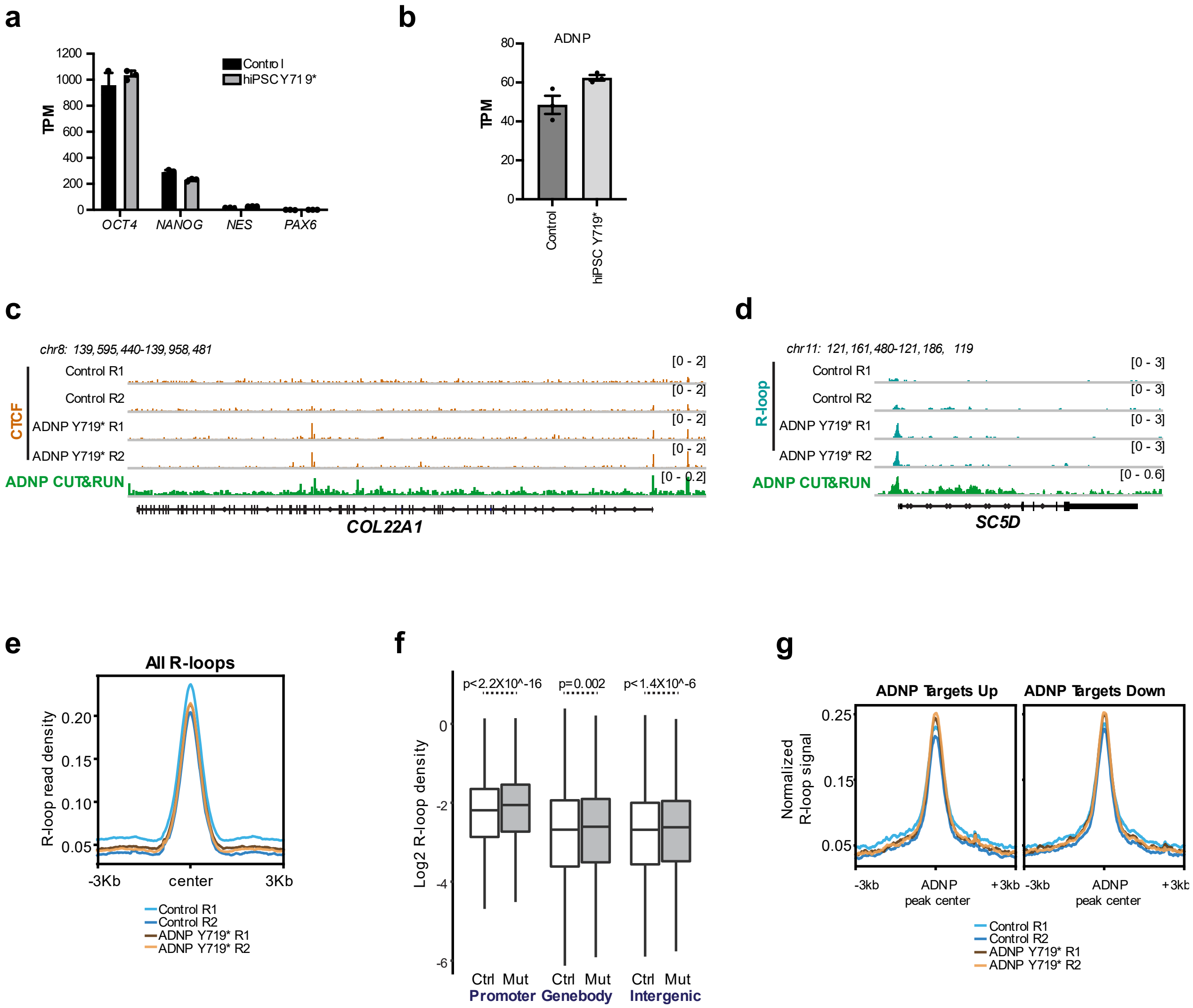

Supplementary Figure 8: R-loops are increased at ADNP targets in ADNP Y719* hiPSCs.

(a) Bar chart of RNA-Seq TPM showing expression of *OCT4*, *NANOG*, *NES* and *PAX6* genes in control and ADNP Y719* hiPSCs. Bar chart, mean + SEM; individual values shown as dots (n = 3 biologically independent samples). Source data are provided as a Source Data file.

(b) Bar chart of RNA-Seq TPM showing expression of *ADNP* gene in control and ADNP Y719* hiPSCs. Bar chart, mean + SEM; individual values shown as dots (n = 3 biologically independent samples). Source data are provided as a Source Data file.

(c) Genome browser view of the full *COL22A1* gene showing CTCF CUT&RUN signal (RPM) in control and ADNP Y179* hiPSCs and ADNP CUT&RUN signal (RPM) in control hiPSCs.

(d) Genome browser view of the full *SC5D* gene showing MapR signal (RPM) in control and ADNP Y179* hiPSCs and ADNP CUT&RUN signal (RPM) in control.

(e) Signal plot of normalized MapR signal in control and ADNP Y719* hiPSCs over 50,692 R-loop MapR peaks called in control and mutant hiPSCs that do not overlap ADNP peaks.

(f) Boxplot displaying log2 normalized R-loop read densities in control and ADNP Y719*hiPSCs across ADNP peaks, grouped by genomic feature. p, Welch's two-sided t-test (n = 12,229 promoter peaks, 11,823 genebody peaks, or 11,768 intergenic peaks). Box, 25th percentile – median – 75th percentile. Whiskers extend to 1.5x interquartile range; outliers not displayed.

(g) Signal plot of normalized MapR signal over ADNP peaks associated with ADNP target genes that are upregulated (4,775 peaks) or downregulated (5,945 peaks) in ADNP Y719* relative to control.

Supplementary Table 1: Primers and oligonucleotides used in this study.

Primer name	Sequence (5'-3')
**Cloning**	
TurboID-BamHI-F	TGCGGATCCATGAAAGACAATACTGTGCCTCTGA
TurboID-Flag-XhoI-R	CCCTCGAGTTATTACTTGTCGTCATCGTCCTTGTAGTCCTTTTCGGCAGACCGC
RNaseH-KpnI-F	GGGGTACCATGCTGAAACAGGTGGAAATCT
RNaseH-BamHI-R	TGCGGATCCCACTTCCACCTGGTAGCCGGTA
DDX5-BamHI-F	TGCGGATCCATGTCGGGTTATTCGAGTGACC
DDX5-Flag-SalI-R	GCGTCGACTTACTTATCGTCGTCATCCTTGTAATCTTGGGAATATCCTGTTGGC
hADNP-SacI-F	CGAGCTCATTTCCAACTTCCTGTCAACAA
hADNP-SpeI-R	GGACTAGTTGGGCCTGTTGGCTGCTCAGTTTA
hADNP-Zn_SpeI-R	GGACTAGTTGATCTAACTTTCGTTTTTTCAG
ADNP-Flag-SacI-F	CGAGCTCATGGACTACAAAGACGATGACGACAAGTTCCAACTTCCTGTCAACAA
ADNP-SalI-R	CGTCGACGGCCTGTTGGCTGCTCAGTTTA
ADNP-HD-BamHI-F	TGCGGATCCTTAGACCCCAAGGGTCATGA
ADNP-HD-SalI-Flag-R	GCGTCGACTTACTTATCGTCGTCATCCTTGTAATCATCACGGACACACTTCTTCC
ADNP-KO-gRNA1-F	CACCGACCCTCTCTACGAGATCGTC
ADNP-KO-gRNA1-R	AAAC GACGATCTCGTAGAGAGGGTC
ADNP-KO-gRNA2-F	CACCGTACCGAAGGTTATTCCGGA
ADNP-KO-gRNA2-R	AAACTCCGGAATAACCTTCGGTAC
ADNP-KI-gRNA3-F	CACCGCATATGCTATGGCACGCCA
ADNP-KI-gRNA3-R	AAACTGGCGTGCCATAGCATATGC
ADNP tag KI donor-left (gBlock)	GACCCAAGCTTGGTACCGAGCTCGGCATATGCTATGGCACGCCAGGGAGTGAGGTAGACCAAGATGATGTAGTTGAGTGGAAAGATGGTGCTTCACCATCTGAGAGTGGGCCTGGTTCCCAACAAATCTCAGACTTTGAGGATAATACATGTGAAATGAAACCAGGAACCTGGTCTGATGAGTCTTCCCAGAGTGAAGATGCAAGGAGCAGTAAGCCAGCTGCCAAAAAAAAGGCTACAGTGCAAGATGACACAGAGCAGTTAAAATGGAAGAATAGTTCCTATGGAAAAGTTGAAGGGTTTTGGTCCAAGGACCAGTCACAGTGGGAAAATGCATCTGAGAATGCAGAGCGCTTACCAAACCCACAGATTGAGTGGCAGAATAGCACAATTGACAGTGAGGACGGGGAGCAGTTTGACAGCATGACTGACGGAGTTGCTGATCCCATGCATGGCAGCTTAACTGGAGTGAAGCTGAGCAGCCAGCAAGCC
ADNP HAV5 donor-insertion (gBlock)	TTAACTGGAGTGAAGCTGAGCAGCCAGCAAGCCTACCCATACGACGTCCCAGACTACGCTGGCAAGCCCATCCCCAACCCCCGCTGGGCCTGGACAGCACCTGAGGCTCTAGAGTGCACTAGCATATGCATATGGGCCGTGTTGCATCCTGGACTTCTGCTCTCCTT
ADNP tag KI donor-right (gBlock)	ATATGGGCCGTGTTGCATCCTGGACTTCTGCTCTCCTTCCAGTCTGACTGCAAAGCTGTCTTCTAACTGGCACTACCTTGCAAGGACTGGTCAGTCAGCAGGCTGTGGGGATGTGTGACCACTGTAGTCTCAGTGGTTATTTCCAAGTCTATGATAGATGACTGGTTGATCTTTGTTCAGACTCTTCTCTGGTAGACTCGGTGATGAAGTGTGAAAAACCAGTAAGCTGGTAGCTCATGAATGCACACAGGGAGAAGTAGTTCCTTACCTACCTGCCTTCTCAGCATTTCTTCCCCTCAGATGTTTGGTTGGATGTCCTGGGAAAGCCTTATCCTGATTCACATAGTAGTGCAGGGCGGGCATACACACTACCAGTCAAATGTGTCTAGTAGACTTGGGAACATGGACCGTTTTTTCATGTATCTTCTGAATAGTTGAAATGTAGCATATGCTATGGCACGCCAGGGATCCATCACACTGGCGGCCGCTCG
ADNPΔHD-gRNA-F Δ	CACCGACAAACAGCCCTATCCCACC

ADNPΔHD-gRNA-R	AAACGGTGGGATAGGGCTGTTTGTC
ADNPΔHD-donor-left (gBlock)	GACCCAAGCTTGGTACCGAGCTCGACAAACAGCCCTATCCCACCAGGGC AGTGCCCTATAAAAAAGATGTTGGGAAGACCCTTTGCCCTCTTTGCTTTTC AATACTAAAAGGACCCATATCTGATGCACTTGCACATCATTTACGAGAAAG ACACCAAGTTATTCAGACAGTTCATCCGGTTGAGAAAAAGCTAACTTACAA ATGTATCCATTGCCTTGGTGTGTATACTAGCAACATGACAGCCTCAACCAT CACTCTGCATCTAGTCCACTGCAGGGGTGTTGGAAAAACCCAGAATGGC CAGGACAAGACAAACGCACCTTCTCGGCTCAATCAGTCTCCAGGCCTGG CCCCTGTGAAGCGCACGTATGAGCAGATGGAGTTTCCACTGCTAAAAAAG CGGAAGCTGGAGGAGGATGCTGATTCCCCTAGCTGCTTTGAAGAGAAGC CAGAAGAGCCTGTTGTTTTAGCTTTAGACGTCCGCGACTGTGA
ADNPdHD-donor-right (gBlock)	GAAGAGCCTGTTGTTTTAGCTTTAGACGTCCGCGACTGTGAAAAGTACAA GCCTGGTGTGCTGCTAGGTTTTAACATGAAAGAATTAAATAAAGTCAAACA CGAGATGGATTTTGATGCTGAGTGGCTGTTTGAAAATCACGATGAGAAAG ACTCAAGAGTCAATGCTAGCAAGACTGTTGACAAAAAGCATAACCTTGGG AAAGAAGATGATAGCTTCTCAGATAGTTTTGAACATTTGGAAGAAGAATCC AATGGAAGCGGGAGTCCTTTTGACCCTGTCTTTGAAGTTGAGCCTAAAAT TCCCAGTGATAATTTAGAGGAGCCTGTACCGAAGGTTATTCCGGAAGGTG CTTTGGAATCTACAAACAGCCCTATCCCACCAGGATCCATCACACTGGCG GCCGCT
ADNP-CRISPRa-gRNA1-F	CACCGACCTACCCATCTGACAAAAA
ADNP-CRISPRa-gRNA1-R	AAACTTTTTGTCAGATGGGTAGGTC
**qRT-PCR**	
Region1-qRT-F	AACACACTGACTACTGCTCTTC
Region1-qRT-R	TGTCAGGGATTGGTGCTTATC
Region2-qRT-F	TGCCTGCTCTTCCACTATTC
Region2-qRT-R	GGGCGTCAGATCCCATTAC
Region_ctl-qRT-F	AGAGAAATCCCAATAGCGTCAG
Region_ctl-qRT-R	TTCACTATTTCCTTGCCCTGG

Supplementary Table 2: List of antibodies used in this study.

ANTIBODY	SOURCE	IDENTIFIER
Monoclonal ANTI-FLAG M2 antibody	Millipore Sigma	F1804
TOP1 antibody	BIO-RAD	VMA00359
ATRX antibody (H-300)	Santa Cruz Biotechnology	Sc-15408
GAPDH (14C10) antibody	Cell Signaling Technology	2118S
Actin antibody	Millipore Sigma	A2066
Anti-α-Tubulin Mouse mAb (DM1A)	Millipore Sigma	CP06
ADNP antibody	Our Lab	
ADNP antibody	R&D Systems	AF5919
ADNP antibody	BETHYL Laboratories	A300-104A
EZH2 antibody	BD Transduction Laboratories	612667
V5-Tag (D3H8Q) antibody	Cell Signaling Technology	13202S
Anti-ds DNA antibody [3519 DNA]	Abcam	ab17256
Anti-HA (12CA5)	Millipore Sigma	11583816001
Rabbit IgG	Millipore Sigma	I5006
Rabbit anti-mouse IgG	Thermo Scientific	SA5-10192
Anti-rabbit IgG (H+L)	Cell Signaling Technology	5151S
Anti-mouse IgG (H+L)	Cell Signaling Technology	5257S
Anti-Goat IgG Secondary Antibody	LI-COR	92532214
S9.6 antibody	Our Lab	
CTCF antibody	Cell Signaling Technology	3418S
Streptavidin-HRP	Cell Signaling Technology	3999
