# Supplementary material for: Proximity labeling identifies a repertoire of site-specific R-loop modulators
Source: Nat Commun. 2022 Jan 10;13:53. doi: 10.1038/s41467-021-27722-6 (PMC8748879; doi:10.1038/s41467-021-27722-6)
Supplement: Supplementary file 2 — Description of additional Supplementary File [file 41467_2021_27722_MOESM2_ESM.pdf]

Description of Additional Supplementary Files

Supplementary Data 1: Mass spectrometry analysis and gene ontology of proteomics data.

Supplementary Data 2: RNA-Seq analysis and gene ontology of WT, ADNP KO, and ADNPΔHD mESCs.

Supplementary Data 3: RNA-Seq analysis and gene ontology of WT and ADNP Y719* hiPSCs.
