# Supplementary material for: Proximity labeling identifies a repertoire of site-specific R-loop modulators
Source: Nat Commun. 2022 Jan 10;13:53. doi: 10.1038/s41467-021-27722-6 (PMC8748879; doi:10.1038/s41467-021-27722-6)
Supplement: Supplementary file 6 — Reporting Summary [file 41467_2021_27722_MOESM6_ESM.pdf]

nature research

Corresponding author(s): Kavitha Sarma

Last updated by author(s): Dec 1, 2021

Reporting Summary

Nature Research wishes to improve the reproducibility of the work that we publish. This form provides structure for consistency and transparency in reporting. For further information on Nature Research policies, see our Editorial Policies and the Editorial Policy Checklist.

Statistics

For all statistical analyses, confirm that the following items are present in the figure legend, table legend, main text, or Methods section.

n/a	Confirmed	
☐	☒	The exact sample size (*n*) for each experimental group/condition, given as a discrete number and unit of measurement
☐	☒	A statement on whether measurements were taken from distinct samples or whether the same sample was measured repeatedly
☐	☒	The statistical test(s) used AND whether they are one- or two-sided<br>*Only common tests should be described solely by name; describe more complex techniques in the Methods section.*
☒	☐	A description of all covariates tested
☒	☐	A description of any assumptions or corrections, such as tests of normality and adjustment for multiple comparisons
☐	☒	A full description of the statistical parameters including central tendency (e.g. means) or other basic estimates (e.g. regression coefficient) AND variation (e.g. standard deviation) or associated estimates of uncertainty (e.g. confidence intervals)
☐	☒	For null hypothesis testing, the test statistic (e.g. *F*, *t*, *r*) with confidence intervals, effect sizes, degrees of freedom and *P* value noted<br>*Give P values as exact values whenever suitable.*
☒	☐	For Bayesian analysis, information on the choice of priors and Markov chain Monte Carlo settings
☒	☐	For hierarchical and complex designs, identification of the appropriate level for tests and full reporting of outcomes
☐	☒	Estimates of effect sizes (e.g. Cohen's *d*, Pearson's *r*), indicating how they were calculated

Our web collection on statistics for biologists contains articles on many of the points above.

Software and code

Policy information about availability of computer code

Data collection	Illumina Basespace : sequencing and base calls
Data analysis	MaxQuant v1.6.4.0 : mass spectrometry peptide identification<br>Bowtie2 v2.2.9 : MapR/CUT&RUN read alignment<br>STAR v2.7.3: RNA-Seq read alignment<br>RSEM v1.3.3: RNA-Seq count estimation<br>MACS2 v2.2.1 : peak calling<br>deepTools v3.4.1: BigWig generation, read density measurements, metaplots and heatmaps<br>R v3.6.1: R-based data analysis<br>ChIPSeeker v1.20.0: Peak annotation<br>DiffBind v2.12.0: Differential peak calling<br>limma v3.40.6: Differential gene expression<br>edgeR v3.26.8: Differential gene expression<br>Enrichr (https://maayanlab.cloud/Enrichr): Gene ontology

For manuscripts utilizing custom algorithms or software that are central to the research but not yet described in published literature, software must be made available to editors and reviewers. We strongly encourage code deposition in a community repository (e.g. GitHub). See the Nature Research guidelines for submitting code & software for further information.

Data

Policy information about availability of data

All manuscripts must include a data availability statement. This statement should provide the following information, where applicable:

- Accession codes, unique identifiers, or web links for publicly available datasets
- A list of figures that have associated raw data
- A description of any restrictions on data availability

All mass spectrometry raw data generated in this study have been deposited to the MassIVE public repository under accession code MSV000087568 (https://massive.ucsd.edu/ProteoSAFe/dataset.jsp?task=8ae3c02d42bf4dc8a98b67ec63ca13b4) and the ProteomeXchange repository under accession code PXD026473 (http://proteomecentral.proteomexchange.org/cgi/GetDataset?ID=PXD026473). MapR, CUT&RUN, and RNA-Seq sequencing data and processed tracks generated in this study have been deposited in the NCBI GEO database under accession code GSE171401 (https://www.ncbi.nlm.nih.gov/geo/query/acc.cgi?acc=GSE171401). RNA-Seq data for WT mESCs used in this study is available in the NCBI GEO database under accession code GSE160578 https://www.ncbi.nlm.nih.gov/geo/query/acc.cgi?acc=GSE160578).

Field-specific reporting

Please select the one below that is the best fit for your research. If you are not sure, read the appropriate sections before making your selection.

[x] Life sciences [ ] Behavioural & social sciences [ ] Ecological, evolutionary & environmental sciences

For a reference copy of the document with all sections, see nature.com/documents/nr-reporting-summary-flat.pdf

Life sciences study design

All studies must disclose on these points even when the disclosure is negative.

Sample size	No sample size calculations were performed. All sequencing experiments were performed with biological replicates as is the norm in the field.
Data exclusions	No data was excluded from analysis
Replication	MapR and CUT&RUN experiments were performed with two biological replicates. RNA-Seq and TurboID experiments were performed with three biological replicates. All replicates produced similar results.
Randomization	This study does not involve participant groups and therefore randomization was not needed
Blinding	This study does not involve participant groups and therefore blinding was not needed

Reporting for specific materials, systems and methods

We require information from authors about some types of materials, experimental systems and methods used in many studies. Here, indicate whether each material, system or method listed is relevant to your study. If you are not sure if a list item applies to your research, read the appropriate section before selecting a response.

Materials & experimental systems

n/a	Involved in the study	
[ ]	[x]	Antibodies
[ ]	[x]	Eukaryotic cell lines
[x]	[ ]	Palaeontology and archaeology
[x]	[ ]	Animals and other organisms
[x]	[ ]	Human research participants
[x]	[ ]	Clinical data
[x]	[ ]	Dual use research of concern

Methods

n/a	Involved in the study	
[ ]	[x]	ChIP-seq
[x]	[ ]	Flow cytometry
[x]	[ ]	MRI-based neuroimaging

Antibodies

Antibodies used

Monoclonal ANTI-FLAG M2 antibody: Millipore Sigma F1804
TOP1 antibody: BIO-RAD VMA00359
ATRX antibody (H-300): Santa Cruz Biotechnology Sc-15408
GAPDH (14C10): antibody Cell Signaling Technology 2118S
Actin antibody: Millipore Sigma A2066
Anti-α-Tubulin Mouse mAb (DM1A): Millipore Sigma CP06
ADNP antibody: Our Lab

	ADNP antibody: R&D Systems AF5919<br>ADNP antibody: BETHYL Laboratories A300-104A<br>EZH2 antibody: BD Transduction Laboratories 612667<br>V5-Tag (D3H8Q) antibody: Cell Signaling Technology 13202S<br>Anti-ds DNA antibody [3519 DNA]: Abcam ab17256<br>Anti-HA (12CA5): Millipore Sigma 11583816001<br>Rabbit IgG: Millipore Sigma I5006<br>Rabbit anti-mouse IgG: Thermo Scientific SA5-10192<br>Anti-rabbit IgG (H+L): Cell Signaling Technology 5151S<br>Anti-mouse IgG (H+L): Cell Signaling Technology 5257S<br>Anti-Goat IgG Secondary Antibody: LI-COR 92532214<br>S9.6 antibody: Our Lab<br>CTCF antibody: Cell Signaling Technology 3418S<br>Streptavidin-HRP: Cell Signaling Technology 3999
Validation	All commercial antibodies were validated for the species and applications for which they were used in this study, and used according to manufacturer's instructions and recommended dilutions. ADNP antibody was extensively characterized and validated for recognition in both human and mouse, as detailed throughout figures in the present study.

Eukaryotic cell lines

Policy information about cell lines

Cell line source(s)	HEK293 cells : Gardini Lab, Wistar Institute<br>SF9 cells: Expression Systems<br>E14 mESCs : Bonasio Lab, University of Pennsylvania<br>PBMC2-iPS4F8 hiPSCs : Real Lab, University of Granada (https://pubmed.ncbi.nlm.nih.gov/31035039)<br>GENYOi004-A hiPSCs : Real Lab, University of Granada (https://pubmed.ncbi.nlm.nih.gov/31035039)
Authentication	Knockout clones were confirmed by Western blot
Mycoplasma contamination	Cell lines tested negative for mycoplasma contamination
Commonly misidentified lines<br>(See ICLAC register)	No cell lines used are listed in the database of commonly misidentified cell lines

ChIP-seq

Data deposition

☒ Confirm that both raw and final processed data have been deposited in a public database such as GEO.

☒ Confirm that you have deposited or provided access to graph files (e.g. BED files) for the called peaks.

Data access links<br>*May remain private before publication.*	https://www.ncbi.nlm.nih.gov/geo/query/acc.cgi?acc=GSE171401
Files in database submission	FASTQ, BigWig, RNA-Seq gene counts
Genome browser session<br>(e.g. UCSC)	NA

Methodology

Replicates	Biological replicates were sequenced for each sample
Sequencing depth	Approximately 15-25 million reads were obtained for each sample. Samples are paired end 75bp
Antibodies	ADNP C-terminal antibody : This study<br>Anti-HA (12CA5) : Millipore Sigma 11583816001<br>Rabbit IgG : Millipore Sigma I5006<br>CTCF : Cell Signaling Technology 3418S
Peak calling parameters	Peaks were called for each sample using MACS2 2.2.182 and parameters "--broad --broad-cutoff 0.1 -f BAMPE -g mm/hs --keep-dup all" for MapR and "-f BAMPE -g mm/hs --keep-dup all" for CUT&RUN
Data quality	Peaks with $p < 0.1$ for MapR and $p < 0.05$ for CUT&RUN and not across an ENCODE blacklist region were considered
Software	Reads were aligned to the mouse reference genome mm10 or human reference genome hg19 using Bowtie2 version 2.2.9 with default parameters
